# TCNQ and Its Derivatives as Electrode Materials in Electrochemical Investigations—Achievement and Prospects: A Review

**DOI:** 10.3390/ma17235864

**Published:** 2024-11-29

**Authors:** Tetiana Starodub, Slawomir Michalkiewicz

**Affiliations:** Institute of Chemistry, Jan Kochanowski University, Uniwersytecka St. 7G, PL-25406 Kielce, Poland; smich@ujk.edu.pl

**Keywords:** 7,7′,8,8′-tetracyanoquinodimethane, radical anion salts, voltammetry, electrochemical (bio)sensors, electrode materials, metal/covalent–organic frameworks, sustainable batteries

## Abstract

7,7′,8,8′-tetracyanoquinodimethane (TCNQ) is one of the most widely used effective surface electron acceptors in organic electronics and sensors, which opens up a very interesting field in electrochemical applications. In this review article, we outline the historical context of electrochemically stable selective electrode materials based on TCNQ and its derivatives and their development, their electrochemical characteristics, and the experimental aspects of their electrochemical applications. TCNQ-modified electrodes are characterized by long-term stability, reproducibility, and a low detection limit compared to other sensors; thus, their use can increase determination speed and flexibility and reduce investigation costs. TCNQ and its derivatives can also be successfully combined with other detector materials for cancer-related clinical diagnostic testing. Examples of simple, rapid, and sensitive detection procedures for various analytes are provided. Applications of new electrochemically stable TCNQ-based metal/covalent–organic hybrid frameworks, with exceptionally large surface areas, tunable pore sizes, diverse functionality, and high electrical conductivity, are also presented. As a result, they also offer enormous potential as revolutionary catalysts, drug carrier systems, and smart materials, as well as for use in gas storage. The use of TCNQ compounds as promising active electrode materials in high-power organic batteries/energy storage devices is discussed. We hope that the information featured in this review will provide readers with a good understanding of the chemistry of TCNQ and, more importantly, help to find good ways to prepare new micro-/nanoelectrode materials for rational sensor design.

## 1. Introduction

**Figure 1:**
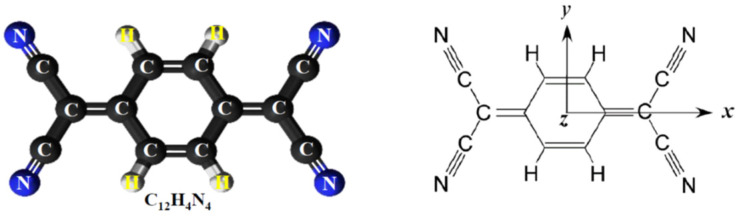
The molecular structure of 7,7′,8,8′-tetracyanoquinodimethane (TCNQ).

Electrochemical methods based on charge exchange reactions occurring at electrode–solution interfaces are very promising tools for obtaining analytical information about examined material objects. Electrochemical sensors measure the (mainly potential and/or current) changes that occur during the interaction of analytes with the sensing surface of the working electrode. These changes can be related to the qualitative and quantitative composition of the analyzed material. For example, information about heterogenous electrochemical activity in biological systems and energy materials can be obtained and studied. Due to the appropriate selection of the working electrode material and solution composition, electrochemical analysis is specific, simple, and reliable, characterized by a low detection limit and a wide dynamic concentration range. To increase their selectivity and sensitivity, sensors are often modified with various inorganic and organic compounds. Recently, there has been growing interest in the use of 7,7′,8,8′-tetracyanoquinodimethane (Figure 1) and its derivatives as electrode surfaces’ modifiers.

**Figure 2:**
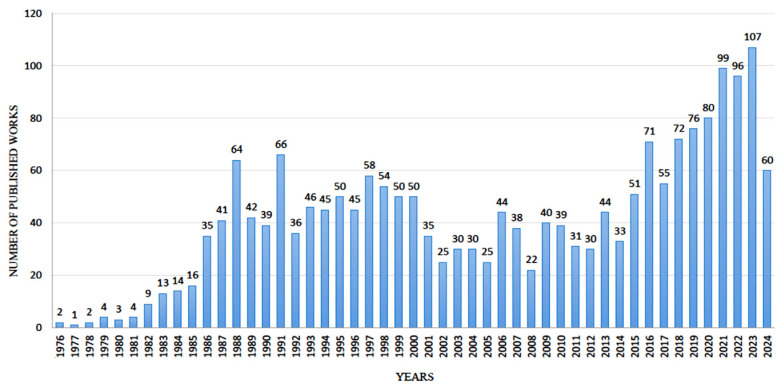
The number of published works on the application of TCNQ and its derivatives in electrochemistry from 1976 to September 2024.

Many scientific and industrial centers around the world are conducting intensive research on the use of these unusual and versatile compounds. This is evidenced by the constantly increasing number of published works on their electrochemical applications, as summarized in the diagram in Figure 2.

The first mention of TCNQ electrochemistry appeared in 1976, and since the 1990s, the number of articles on the topic has steadily increased. In just one case, using ScienceDirect, one of the world’s largest collections of research articles and books available on ELSEVIER, a database with a “search within article title, abstract, keywords” option, 2220 results appeared for the entries “TCNQ electrode materials” and “electrochemistry of TCNQ” (as of 26 September 2024). In recent years, the construction of modified electrodes based on TCNQ and its derivatives has become one of the dominant methods for obtaining sensors used in many research areas. Currently, TCNQ-based electrodes enable the determination of an increasing number of analytes while demonstrating progressively improved performance parameters.

**Figure 3:**
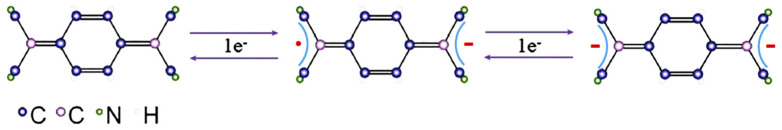
Two-electron reversible reaction of TCNQ.

TCNQ has been known as an organic molecule with unusual properties of p-electron acceptors [1,2] and the stability of negatively charged species. This redox-active molecule can exist in two forms: the TCNQ^•−^ radical anion and the TCNQ^2−^ dianion in acetonitrile and 1,2-dichloroethane medium (Figure 3) [3,4,5,6].

The chemistry and electrochemistry of TCNQ and its redox forms have been poorly explored [5]. Although TCNQ is insoluble in water, it is possible to study its interactions with aqueous electrolytes by the means of liquid/liquid electrochemistry using scanning electrochemical microscopy (SECM) [7]. For example, Ref. [8] showed that TCNQ reduction at a three-phase junction between water, 2-nitrophenyl octyl ether, and a screen-printed carbon electrode (SPCE) can lead to the transfer of cations from the aqueous to the organic phase.

**Scheme 1:**
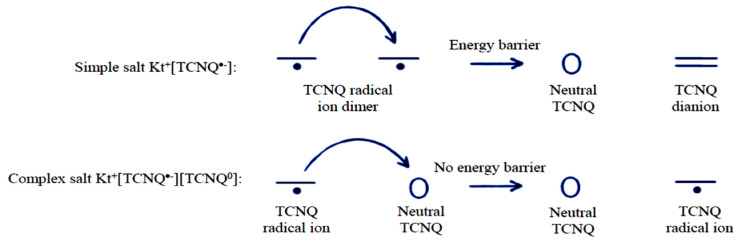
The formation of two series of TCNQ salts: Kt^+^[TCNQ^•−^], simple salts, and Kt^+^[TCNQ^•−^][TCNQ^0^], complex salts.

Anion radical salts (ARS) formed by TCNQ attracted researchers’ attention as early as the mid-1960s [1,2]. Later, in 2014, the unique properties of both ARS and charge-transfer complexes (CTCs) formed by TCNQ as strong p-electron acceptors were described in detail [9]. Many papers have been published on TCNQ ARS synthesis. Generally, controlling the molar proportion of the starting materials is a semi-empirical way to obtain organic CTCs with different stoichiometries. In combination with a variety of cations, owing to its high electron affinity (*A* = 2.8 or 3.0 eV; [10,11]), the TCNQ^•−^ radical anion can form two series of salts with the compositions Kt^+^[TCNQ^•−^] (the simple salts) and Kt^+^[TCNQ^•−^][TCNQ^0^] (the complex salts), in which Kt^n+^ may be an organic or metal cation [9]. With the simple salts, in order for an electron to move along the stack, coulombic forces have to be overcome to form a dianion. When TCNQ^0^ is added, as in the complex salts, higher conductivity is obtained (Scheme 1). Therefore, the use of heterocyclic compounds as cations is an important factor in the formation of multifunctional materials.

The ability to form the stable anion radical enables the synthesis of highly conductive TCNQ ARS of an intricate composition. The conductivity of simple salts is generally substantially lower than that of complex salts and is attributed to TCNQ molecules. The mode of conduction is considered electronic. Salts of an intricate composition contain TCNQ species with a charge (*q*) of 0 < *q* < −1, i.e., the crystals of these ARS formally contain both TCNQ molecules and TCNQ^•−^ radical anions. These include, for example, the anion radical salts Cs_2_(TCNQ)_3_ and QnH(TCNQ)_2_ (QnH^+^ is a quinolinium cation), in which the oxidation state of TCNQ is −2/3 and −1/2, respectively.

The first conductive organic ARS materials with chemical stability were obtained and described in the mid-1980s [12,13]. Later, it was found that ARS based on TCNQ are able to form spin ladders, ferromagnetic structures, and other magnetically ordered structures under normal conditions [14,15,16]. This has opened up great opportunities for the use of TCNQ ARS as potential materials in various electronic industries including spintronics and non-linear optics [17,18]. Not surprisingly, interest in TCNQ-based compounds has grown significantly.

**Figure 4:**
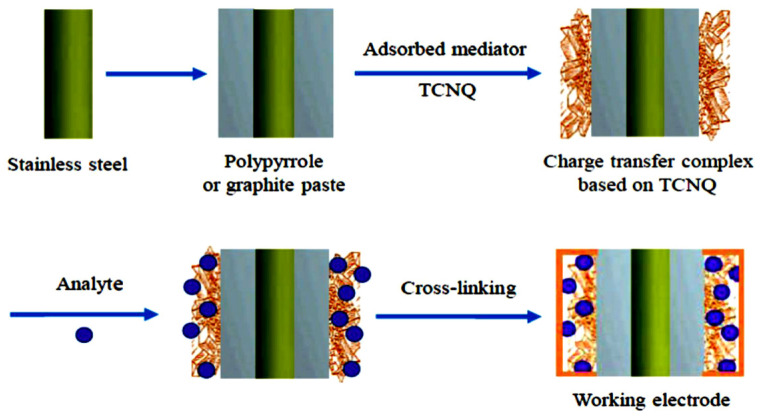
General scheme for obtaining electrode mediators based on TCNQ or its CTC.

Electrochemical techniques have a long history of being used as analytical tools and for material synthesis in the various fields of electroanalysis and electrosynthesis [18]. There are three electrical changes in electroanalysis: conductometric (a change in the ability of the sensing materials to transport charge), potentiometric (a change in the measured voltage between the reference electrodes and indicator), and amperometric (a change in the measured current at a given applied voltage) changes. Thus, these techniques hold great promise for a large field of use, including the fabrication of miniature implantable biosensors, the monitoring of the results of analytical tests, and medical diagnosis. Nicholson and Shain, in [19], described theoretical ideas, models, and chemical reactions on the topic of cyclic voltammetry (CV). CV is an experimental electrochemical technique that allows for the determination of different kinetic parameters, and, therefore, it generates huge interest. The π-system of TCNQ ARS allows the exploration of the electron-accepting abilities of this class of compounds [20,21]. According to research on the π-system based on CV data, TCNQ derivatives are poorer electron acceptors than TCNQ [22,23,24], and the TCNQ CTC shows excellent electrocatalytic properties. Therefore, in the past two decades, TCNQ and its CTC have received great interest for the construction of electrochemical sensors with a wide range of possibilities (Figure 4). They include, for example, electrochemical glucose biosensors [25,26,27,28], amperometric cholesterol biosensors [29], and biosensors for monitoring formaldehyde [30] based on TCNQ surface modifiers. These sensors exhibit desirable properties such as composition-dependent charge mobility, 1D morphology, and air stability [31,32]. TCNQ mediators lower the limit of detection (LOD) of biosensors, opening ways to new, exciting applications [33]. In addition, TCNQ and its ARS and CTC have attracted interest since they are quite easily synthesized; this allows the obtainment of compounds based on TCNQ with different crystal structures and physicochemical properties.

In recent years, new horizons for the application of nanomaterials in bioelectroanalytical chemistry have opened. Many nanomaterials based on graphene, fullerene, metals, and porphyrin (an essential compound for life on Earth) and its derivatives are already used to construct electrochemical sensors [34,35,36,37,38,39]. Graphene is especially a very promising material for nanoelectronics and nanotechnologies [39,40,41,42]. Novoselov and co-workers [43] were the first to investigate graphene’s properties. In 2004, this research group described that TCNQ can be used for the functionalization of graphene. TCNQ can promote effective electron delocalization within the graphene lattice, thus increasing the electrical conductivity of graphene. This is possible thanks to the perfect donor and acceptor properties of graphene. In recent years, TCNQ solution has successfully been used in the production of graphene from graphite too [44,45,46,47]. TCNQ-anion-stabilized graphene sheets have been extensively used in biosensor performance and as a promising candidate for transparent conducting electrode application. This is due to its desirable properties such as an increase in the electroactive surface area of the working electrode, an increase in its sensitivity, and, thus, a decrease in the LOD [48,49,50,51]. However, the transmittance of graphene/TCNQ electrodes decreases with an increase in the number of layers. Another well-known example with a widespread use for improving the performance of graphene transparent conductive electrodes (TCEs) for various devices is a very strong electron acceptor: tetrafluoro-tetracyanoquinodimethane (F_4_TCNQ) [51,52,53]. Currently, TCEs are the key component in photovoltaic technology [54].

**Figure 5:**
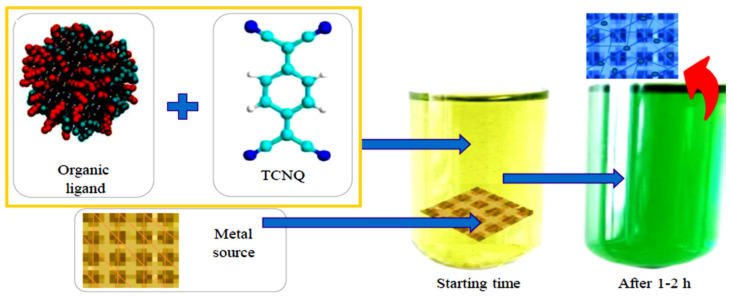
Schematic illustration of the formation of MOF based on TCNQ.

Among various redox-active dopants, TCNQ improves the charge transfer of various materials including those in metal–organic frameworks (MOF) [55,56]. TCNQ as strongly acceptor receives great interest for the construction of modified MOF with a wide range of possibilities (Figure 5). These frameworks are a relatively new perspective class of porous materials which have a high surface area and structural tailorability in a crystalline state [57,58,59,60,61,62]. As in the case of graphene, TCNQ within MOFs significantly improves their electrical conductivity. Frameworks modified by TCNQ show a significantly high redox activity too [63,64,65,66,67,68].

TCNQ and its ARS can also be used as cathode materials in aqueous batteries [69,70]. More than 20 years ago, it was discovered that TCNQ molecules could be used for inorganic cation transfer in a thin-film configuration [71]. In 2011 and 2019, the applicability of TCNQ for cation-coupled electron transfer in a three-phase junction setup [8,72] was investigated.

**Figure 6:**
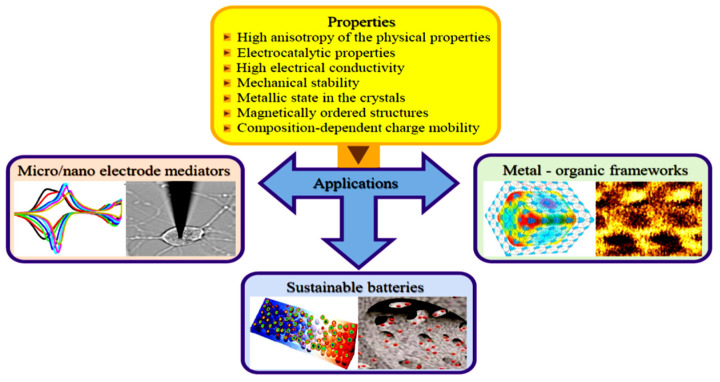
Three major applications of TCNQ and its derivatives in electrochemical techniques.

In summary, the properties of TCNQ and its derivatives, in particular its anionic radical salts, enable their use in electrochemical techniques in three main areas (Figure 6):

1. Micro-/nanoelectrode mediators in electrochemical biosensors.
2. TCNQ-doped metal–organic frameworks as electrode materials.
3. Organic small molecular electrodes in sustainable batteries.

In this review, we present important examples of the three types of applications. We intend to highlight the role of this class of compounds in the development of the electroanalytical roots of TCNQ and other TCNQ-containing compounds in electrochemical and biological sciences. There are other articles or reviews that cover the applications of compounds based on TCNQ. However, all of them relate to their use in electronics and spintronics as organic conductors and semiconductors [1,2,9,17,20,73,74], ferroelectrics [9,14,15,20,73,74,75], spin ladders [6,15], components of magnetic MOFs [76,77,78,79], and materials for non-linear optics [80]. We believe that this review will help to discover novel TCNQ-containing compounds for the production and study of new electrode materials that will be usable in electrochemical investigations and the construction of batteries for energy storage.

## 2. Electrical Properties of TCNQ and Its Conductive TTF-TCNQ Derivative

The electrical properties of TCNQ in the presence of alkali metal ions have been well studied. Studies of the microcrystals of TCNQ immobilized on electrodes in aqueous electrolytes have shown that alkali cations readily form salts with TCNQ^•−^ anion radicals [71,81,82]. The highest solubility has been observed for Li^+^TCNQ^•−^, whereas other alkali salts, as well as those formed with bivalent cations, are much less soluble [81]. The theory of ion transfer at a three-phase electrode has recently been summarized by Scholz and co-workers in [83]. The marked decrease in peak currents for subsequent CV cycles indicated the removal of the redox probe during cycling. This was probably due to an increase in the water solubility of TCNQ upon salt formation between TCNQ anion radicals formed during reduction at a three-phase junction and alkali metal cations. TCNQ anion treating with metal iodides, certain metals, or alkyl-substituted ammonium very easily formed TCNQ ARS of alkaline earth metal, alkali, or tetraalkylammonium ions at room temperature [1,84]. This led to the conclusion that the electrochemical applications of TCNQ depend on many factors and are very complex.

**Figure 7:**
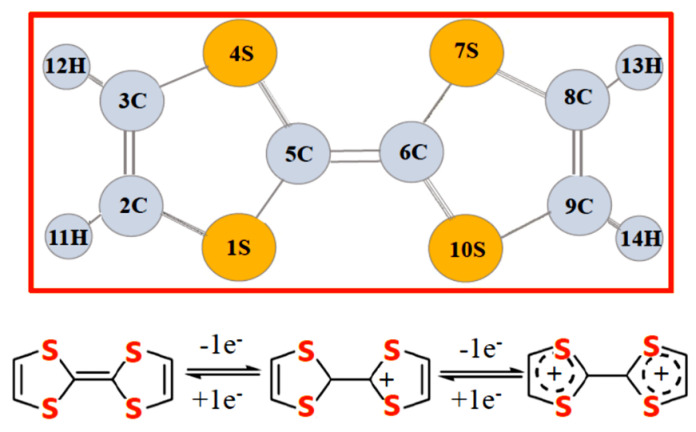
The optimized structure of a tetrathiafulvalene molecule as superconductor and the scheme of TTF oxidation.

The best class of materials extensively studied on micro-/nanoelectrodes are tetrathiafulvalene (TTF)-based conductive charge-transfer salts, including those of TCNQ [85,86,87,88,89,90]. The TTF p-electron donor and TTF derivatives have recently emerged as key constituents for new applications, for example, as molecular sensors, logic gates, redox-fluorescent switches, or molecular clips [88]. TTF itself has unusual electrical properties. Electrochemical measurements show that it can be oxidized in acetonitrile in two successive reversible reactions, yielding a radical cation (TTF^+^) and a dication (TTF^2+^), respectively (Figure 7).

The TTF-TCNQ charge-transfer complex is considered the first synthetic organic metal and has been of great interest to solid-state physicists and physical chemists since its synthesis in 1973 [89]. The first use of the complex as an electrode material was described in 1979 by Jaeger and Bard [90], but the analytical utility of conductive salts was only demonstrated when Kulys oxidized glucose on unmodified organic metal electrodes in 1980 [91]. The works of the second half of the 20th century have shown the broad applications of TTF-TCNQ to bioanalysis [92,93,94]. This organic complex is also interesting as an electrode material for the oxidation of enzymes at low overpotentials [95,96]. Pioneering works in this field were presented by Kulys and co-workers, with other organic salt complexes of N-methylphenazinium (NMP^+^) or N-methylacridinium (NMA^+^) cations with TCNQ [91,97]. Kulys et al. first reported the use of conductive salts in redox reactions with enzymes [91,98]. NMP-TCNQ, NMP(TCNQ)_2_, and NMA-TCNQ as electrode materials in the form of a paste or pressed pellets with glucose oxidase, lactate dehydrogenase, peroxidase, and xanthine oxidase, respectively, was used.

In 1997, Bartlett and Tong [99] reported dissolution phenomena on TTF-TCNQ single-crystal electrode surfaces studied using electrochemical scanning tunneling microscopy (ECSTM). In the range from about −0.2 to +0.4 V vs. SCE, only nonfaradaic capacitive currents were observed. If the electrode potential was taken to be a value more negative than −0.2 V, the TTF-TCNQ was reduced to give TTF^0^ and TCNQ^2−^ dianions [100]. These phenomena could be explained by the anisotropic behavior of the material [99]. The partial charge transfer and very strong intermolecular bonding between the molecules in molecular stacks was observed, whereas these interactions between the stacks were much weaker. TTF-TCNQ can be used for the immobilization of enzymes. This is possible thanks to the regular porous structure of TTF-TCNQ. TTF-TCNQ forms a highly irregular porous structure, which serves as a matrix for the immobilization of enzymes. This underlines the importance of optimizing the enzymatic electrode architecture. TCNQ species can also act as mediators, but only in a more positive potential range.

Currently, mediator structures based on TTF-TCNQ’s or TCNQ’s thin monolayers are especially attractive as they are more effective for electroanalytical applications compared to thick layers. For example, a TTF-TCNQ monolayer can be deposited using thiol-functionalized TTF (TTF-CH_2_SH) with TCNQ on Au(111). The formed films are slightly thicker than the layer-by-layer adsorber ones due to different film structures [101,102]. TTF-TCNQ electrodes behave as conventional metallic electrodes within their stable potential range [103]. TTF-TCNQ is also an efficient and stable component of paste electrodes. They have been tested voltametrically using a rotating disk electrode (RDE) in a neutral phosphate buffer. This environment had a convenient potential window (from −0.50 to +0.25 V vs. Ag/AgCl) for directly studying the reaction of small molecules, for example, hypoxanthine and lactate [104], for in vivo measurements [105,106] as well as for the electrocatalytic oxidation of catechol [103], ascorbic acid (AA) [107,108,109], nicotinamide adenine dinucleotide β-NAD^+^ [110,111,112,113], dopamine [114], and NO_2_ sensing [115]. The TTF-TCNQ electron-transfer complex is also used as an electrode material for flavoenzyme regeneration [94]. It has also been used to develop various biosensors [116]. For example, TTF-TCNQ can be used as a sensor to determine reduced glutathione at a potential of +0.20 V vs. SCE with a detection limit of 4.2 mmol L^−1^ [117].

## 3. Highly Efficient Biosensors Based on TCNQ and TTF-TCNQ

The interesting properties of TCNQ and its derivatives have led to the recent publications of many promising studies related to their use in electroanalysis, as well as in electronic and optoelectronic devices. Glucose oxidase (GOx) has been recognized as an efficient and the most popular choice for enzymatic glucose biosensors due to its high surface charge density, high selectivity, good sensitivity, and fast response [118,119]. For this reason, this compound is the most common enzyme for electrochemical investigations [21,95,103,120,121,122,123,124]. The excellent electrical properties of TTF-TCNQ are most widely used for glucose development. The glucose concentration value by measuring the potential difference between a Ag/AgCl and TTF-TCNQ reference electrode is influenced by the fact that the high density of positively charged TTF donor states causes the selectivity of negatively charged ions towards the TTF surface.

The controlled level of glucose concentration in blood is a very important life parameter. To fight the prevalence of life-threatening disease, the production of simple glucose biosensors is necessary. These new sensors should have the following characteristics: a good selectivity and sensitivity and very high thermal stability. The first glucose sensors using TCNQ as a mediator were obtained by Hendry and co-authors in 1988 [125]. The team described an amperometric enzyme electrode which utilized a TCNQ mediator to facilitate electron transfer from glucose oxidase to a pyrolytic graphite electrode in a clinically relevant range (0–70 mM). In 1999, Li et al. [27] described an amperometric glucose biosensor, stable for 15–30 days, based on TTF-TCNQ as a mediator, operating at +0.20 V vs. SCE in a stirred air-saturated phosphate buffer (pH 7.0). GOx was immobilized electrochemically together with a polypyrrole (PPy) membrane, while TTF-TCNQ salt was adsorbed in two steps on a platinized platinum wire (PPW). According to the authors, the TTF-TCNQ significantly increased the oxidation of H_2_O_2_. The electrode response current to the ascorbate showed a significant increase from −0.27 to 0.38 and from 0.92 to 1.86 mA, with an increase in the polarization potential from 0.1 to 0.2 and 0.3 to 0.4 V vs. SCE, respectively. This is consistent with earlier observations [26,124].

The qualitatively similar behavior of the TCNQ enzymatic electrode on pyrolytic graphite has been observed [126], which is also comparable to that of a glucose biosensor based on carbon black strips [127]. According to [128], TTF-TCNQ can be used as a mediator for analytical bioelectrochemistry since its redox potential is more positive than that of the enzyme.

TTF, analogous to TCNQ, is also known as a good component of the enzymatic electrode, which can be used as a redox mediator for the regeneration of reduced GOx [124,129]. According to Ivanov et al., the enzymatic electrode based on TCNQ exhibits capacitive behavior by having a potential is near 0 V [124]. On the other hand, a redox process occurs when the potential is increased to 0.2 and 0.4 V for the TTF^0^/TTF^+^ couple, respectively. Additionally, a well-defined peak on CV curves, corresponding to the reduction in TTF^+^ back to TTF^0^, is present in the negative scan. Such behavior is typical for copper. In this case, the dissolution or oxidation of the copper electrode in the positive scan occurs, whereas copper redeposition in the negative scan occurs [130]. Similar electrochemical oxidation behavior has been shown in the case of TTF-modified carbon nanotubes [131]. Due to this, in the past two decades, glucose biosensors based on TTF-TCNQ-GOx biocomposite have been widely used [28,41,105,116].

**Figure 8:**
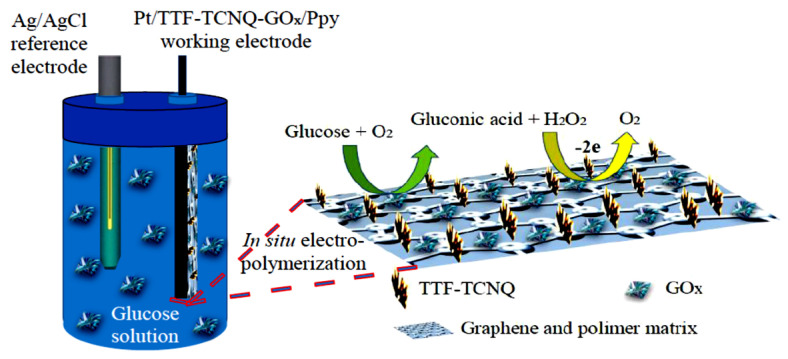
Schematic illustration of a glucose sensor, with functionalizing TTF-TCNQ on the PPW working electrode and the electrochemical processes near the working electrode.

Pandey et al. [132] developed a new electrocatalytic organically modified sol–gel glass (ormosil) material encapsulated with TTF-TCNQ. Recent reviews on the use of sol–gel glass technology have summarized the applications of this technology attributed to enzyme electrodes [133,134]. This ormosil was made using palladium-linked glycidoxypropyltrimethoxysilane TTF-TCNQ powder and a precursor. The amperometric response was recorded at 0.05 V, 0.1 V, and 0.2 V vs. Ag/AgCl. Pandey described the disappearance of the redox peaks of TCNQ or TTF in background cyclic voltammograms and a relatively much larger increase in the anodic current after the addition of glucose compared to the ormosil encapsulated with either TCNQ or TTF. This fact allows us to draw the conclusion that TTF-TCNQ can be used as an electrode mediator for glucose oxidase determination. Figure 8 shows the scheme of a glucose sensor using a platinized platinum wire comprising functionalized TTF-TCNQ or TCNQ and Ag/AgCl as the working and reference electrodes, respectively. Figure 8 also contains a description of the main electrochemical processes near the working electrode.

The above example of a glucose sensor based on TCNQ/TTF-TCNQ exhibits excellent storage stability, as evidenced by a series of repeated experiments for several to several tens of consecutive days. Compared to previous glucose sensors, the new sensors based on TCNQ and TTF-TCNQ can be operated as a single-compartment cell without a membrane between the anode and cathode. Thus, they can be used for routine diagnosis, retaining their sensitivity and reusability for long durations of time.

The results of numerous measurements conducted reveal that TCNQ and TTF-TCNQ have a positive effect on the performance of electrochemical sensors (Table 1). They have been applied to the analysis of many important species such as glutathione [50,117], ascorbic acid [108,109], choline oxidase, betaine [109], adenine [112], nitrogen dioxide gas [115], hydrogen peroxide [128], xanthine [135], heavy metals [136,137], dopamine [138], xanthine oxidase [139], glucose [140,141], sulphite oxidase [142], formaldehyde [143], acetylcholinesterase inhibitors [144], carbamate drugs [145], carbamic and anticholinesterase pesticides [146,147], alkali metals [148], ascorbic acid [149], perphenazine [150], nicotinamide adenine dinucleotide [151], and organophosphorus pesticides [152,153]. In addition, TCNQ and TTF-TCNQ monolayers enhance excellent electrocatalytic properties and have better contact with the active enzyme centers. These monolayers forming on carbon nanotubes have a larger active area and higher response current than planar or fiber electrodes [154]. As a result, lower limits of detection and a wide linear range of concentrations can be achieved. TTF and TCNQ micro-/nanospecies can be generated from the CTC surface either by simple dissolution or by potential induced salt decomposition. Low toxicity is also an important advantage of this type of biosensors. We hope that these data and conclusions will be beneficial for other research and studies employing TCNQ and TTF-TCNQ.

Most of the electrochemical biosensors presented in Table 1 have opened new possibilities for biomarker analysis due to their high sensitivity, good specificity, quick response, and low detection limits. As examples, we would like to draw particular attention to the following references: [155], 2006; [144], 2012; [149], 2023; [150], 2023; and [151], 2024.

L-glutamic acid and monosodium glutamate are responsible for the pleasant taste of food. For example, tomatoes and corn contain large amounts of monosodium glutamate (≅130–140 mg per 100 g of vegetable). In 2006, a very interesting example of an L-glutamate biosensor based on TTF-TCNQ for the quality control of tomato food products was used and described [155]. The TTF-TCNQ paste was used as the working electrode in combination with GLOx on a screen-printed electrode (SPE). The electrochemical oxidation of TCNQ^−^ to TCNQ occurred in the potential range from +0.05 to +0.40 V vs. Ag/AgCl, with TCNQ being reduced to TCNQ^−^ in the reverse potential scan, thus regenerating the enzyme. The authors suggested that the best operating potential of the sensor was in the range of +0.05 to +0.10 V, with an analyte LOD of 0.05 mM. The authors of [155] showed that the TCNQ electrode mediator becomes unstable after long periods of work at lower potentials. The optimum pH for L-glutamic acid and monosodium glutamate activity is about a physiological pH = 7.46. So, a conclusion can be made that a TTF-TCNQ-based L-glutamate biosensor is a perfect stable sensor for the quality control of tomato products. Therefore, this TTF-TCNQ biosensor can be successfully used in the food industry.

The discovery of highly conductive TCNQ paramagnetic salt represented an important advance in the development of biosensors based on acetylcholinesterase (AChE) inhibitors [152]. In 2012, Rotariu et al. studied the use of multi-walled carbon nanotubes (MWCNTs) modified with TCNQ as sensors for the sensitive detection of acetylcholinesterase inhibitors (pesticide residues) [144]. Two AChE inhibitors were used in sol–gel for the construction of a new amperometric acetylcholinesterase biosensor with a very low LOD of 30 pM and 0.4 nM. The authors [153,154] pointed out that this was one of the lowest LODs in electrochemical tests from using AChE. The signal reached 96% of the initial value after 1 week, indicating the good stability of the AChE biosensor. The MWCNT-TCNQ/SPE/AChE/Nafion system, as an example of a nanosensor, will be discussed in detail in the next section. The AChE immobilization procedure can significantly affect the analytical parameters of the biosensor.

In 2023, a novel stable electrochemical sensor based on a TCNQ-modified electrode for the electrochemical sensing of ascorbic acid was developed [149]. The CPE/TCNQ-PdNP sensor demonstrated outstanding properties under optimum conditions (working potential of 0.22 V vs. Ag/AgCl; pH = 7), with a linear response in the range from 50 to 625 mM, detection limits of 51.61, 44.38, and 30.10 μM, and a sensitivity of 0.005, 0.0075, and 0.0148 mA/mM for CPE/TCNQ, CPE/TCNQ-PdNP-1, and CPE/TCNQ-PdNP-2 electrodes, respectively. The TCNQ-modified electrode tended to respond faster in the presence of palladium. A gradual increase in the peak current as a function of palladium nanogeometry was observed.

In the work in [150], MWCNT/[BMIM][PF_6_]-[TTF-TCNQ/IL]/[BMIM][PF_6_] gels for the development of CYP2D6 biosensors were constructed via the physical adsorption of the TTF-TCNQ/IL gel onto the MWCNT/IL gel-premodified SPE with the subsequent electrostatic attachment of CYP2D6. The CYP2D6 biosensor is a promising technology for detecting and determining PPZ, a widely used antipsychotic drug that effectively treats people with schizophrenia and bipolar disorder. The novel TTF-TCNQ/ionic liquid (IL) exhibits both electronic and ionic conductivity. The linearity range and LOD for perphenazine are from 6∙10^−5^ to 1.6∙10^−2^ M and 0.0205 µM, respectively. The MWCNT/IL-[TTF-TCNQ]/IL gel-based CYP2D6 biosensor is expected to open up new possibilities and advance drug analysis.

The work in [151] described the design and validation of a novel, stable, and reproducible electrochemical sensor based on TCNQ and Pd-Co@NC nanocomposite-modified electrodes (TCNQ-Pd-Co@NC/CPE) for the precise and selective sensing of nicotinamide adenine dinucleotide (NADH). The amperometric investigations showed a wide linear concentration range (10–250 µM), with a low LOD of 5.17 µM. The sensor featured a high electron transfer capability and thus sensitivity to NADH.

In conclusion, TCNQ and TTF-TCNQ have a positive effect on the performance of electrochemical sensors. They can be applied as good components of an enzymatic electrode, which can be used as a redox mediator for the analysis of many important species. The excellent physical, chemical, and electrical properties of TCNQ and TTF-TCNQ are widely used for the determination of various analytes. As a result, low limits of detection and wide linear ranges of concentrations can be achieved. The incorporation of mediators based on TCNQ increases the response of biosensors and the sensitivity of electrochemical investigations. TCNQ-modified biosensors are also characterized by long-term stability and reproducibility compared to other sensors. An important advantage of TCNQ/TTF-TCNQ biosensors is also their low toxicity. Their high sensitivity, good selectivity, short response time, and excellent reusability indicate that TCNQ or TTF-TCNQ-based biosensors are affordable tools and promising candidates for the determination of many analytes such as pesticides in environmental monitoring, food ingredients, and drugs in clinical diagnostics. 

## 4. Electrode Nanomaterials Based on TCNQ

Over the past 35 years, the size of electrochemical biosensors has gradually decreased, from devices containing electrodes with micrometer dimensions to nanoelectrodes. Their small size is an advantage because it allows for the less invasive study of biological analytes in small intracellular and extracellular environments compared to larger electrodes. Nanoparticles have also been immobilized to the electrode surface using different chemical surface modification strategies [159,160,161]. The effective electrode size after the attachment of a particle can be estimated by comparing the voltametric limiting currents for the electrochemical reaction of redox mediators, both before and after particle deposition [160,162]. A key advantage of all techniques addressing electrochemistry in single nanoparticles is the extremely fast mass transport of reactants and reaction products to and away from the electrode surface, which allows for studying electrocatalytic reactions with the minimum influence of mass transport limitations. For example, in 2015, a new type of potentiometric solid-state ion-selective electrode (SS-ISE) was fabricated with an intermediate layer made from a TCNQ ionophore-based ion-selective membrane [163]. Paczosa-Bator et al. described the applicability of nanostructured TCNQ to potentiometric ion-selective K^+^ and Na^+^ electrodes. The authors explained the influence of the TCNQ layers on the increase in electrode selectivity and demonstrated the high stability of the potential of the new solid-contact electrodes for the determination of K^+^ and Na^+^ ions. These properties confirmed the possibility of analytical application of SS-ISEs based on TCNQ, influencing the development of a new solid-state ion sensor group.

Surface doping can effectively modify semiconductors and nanostructures at relatively low costs, thus facilitating the development of organic and nanoelectronics. Carbon nanofibers (CNFs) and carbon nanotubes (CNTs) have intriguing structural, electrical, and mechanical properties and therefore attract a wide range of attention [40,164]. CNFs are three-dimensional materials which can accommodate more active materials than a simple two-dimensional structure could hold [39]. Carbon nanotubes are well known for their high surface area, high conductivity, and stability. These variants of graphene have brought progress in the development of a new generation of electrode materials. For example, as mentioned above, Rotariu and co-authors developed a simple, highly sensitive amperometric AChE MWCNTs-TCNQ pesticide biosensor using a carbon electrode modified with carbon nanotubes. This sensor could be combined with other portable devices for on-site measurements. The conductive and magnetic properties of TCNQ salts were important advancements that allowed the construction of effective AChE-inhibition biosensors [151]. Another electroactive compound is enzymatically generated thiocholine (TCh). It too can be used as indicator of pesticides oxidizing on carbon-based electrodes (+0.7 V vs. Ag/AgCl). This high potential can reduce sensitivity toward other electroactive species present in samples. Potassium ferrocyanide [165], cobalt phthalocyanine (CoPC) [166], Prussian blue [167], cobalt hexacyanoferrate [168], and thiocholine [169] can be used as mediators to reduce the oxidation potential of TCh. However, studies on CNTs based on TCNQ have rarely been reported. They only refer to spectroscopic characterizations. The amount of CNT→TCNQ charge transfer is proportional to the CNT diameter, and TCNQ–CNTs binding energy increases as a function of the nanotube radius.

**Figure 9:**
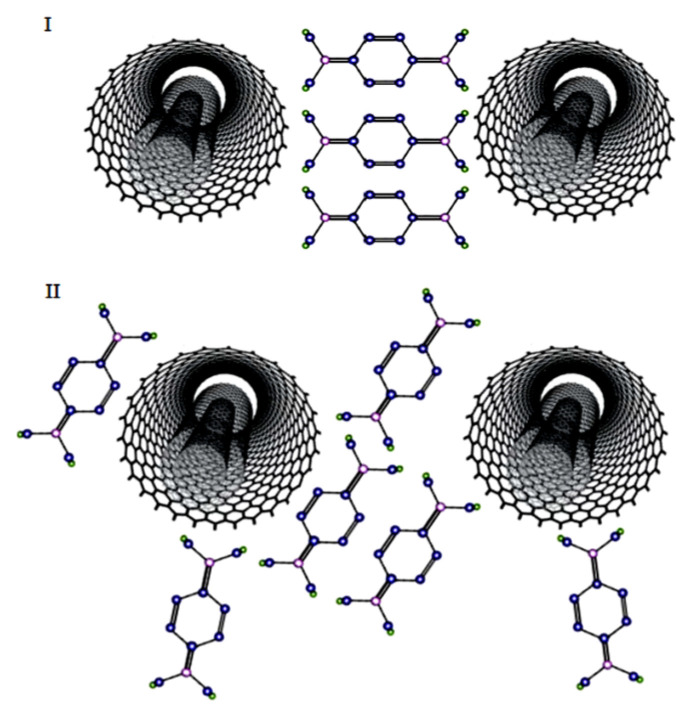
Supramolecular organization of multi-walled CNTs–TCNQ at (**I**) low (<1 mM) and (**II**) high (>10 mM) concentrations of TCNQ.

Veiga and Miwa obtained the most energetically stable adsorption geometries [170]. For zigzag SWCNTs, they mapped the SWCNT→TCNQ electronic charge transfers and examined the electronic structure of the TCNQ/SWCNT system as a function of the SWCNT diameter and chirality. The results revealed that the minimum SWCNT diameter required to exothermically encapsulate a TCNQ molecule was around 0.9 nm. The electronic charge transfers always occurred from the CNT toward the molecule and were proportional to the TCNQ–CNTs binding energy. The authors of [144,151] found and described two different supramolecular arrangements based on different CNTs–TCNQ ratios (Figure 9).

According to Rotariu et al. [144], two oxidation peaks were observed with a higher amount of TCNQ in a phosphate buffer solution (pH: 8). One could be attributed to the MWCNTs–TCNQ and the other to the free or weakly bound TCNQ. The second peak was similar to the one observed for the TCNQ/SPEs sensor. It was observed that the oxidation potential for TCh varied depending on the sensor used, decreasing from +0.70 (bare SPE) to +0.30 V (TCNQ/SPE) and +0.15 V (MWCNTs–TCNQ/SPE). The authors suggested that this fact was attributed to the electron transfer enhancement provided by a synergistic effect between the TCNQ and MWCNTs. The work illustrated that TCNQ and MWCNTs can interact in various ways and form supramolecular arrangements with enhanced electrochemical properties, which can be exploited in biosensor development. Only MWCNT–TCNQ (1 mM)/SPE led to a sensitive, stable, and reproducible response of the TCh sensor. The increased electrocatalytical activity of the MWCNTs–TCNQ was related to the distribution of TCNQ molecules between or along the CNTs and/or additional electrostatic interactions that stabilized the mediator in the MWCNT–TCNQ system [171]. In comparison to other AChE biosensors based on CNTs and/or mediators, the MWCNTs–TCNQ biosensor proposed by Rotariu exhibits very high sensitivity, good reproducibility, and stability. The excellent properties of graphene-based nanomaterials make them suitable for the construction of many biosensors [172,173,174,175,176,177].

**Figure 10:**
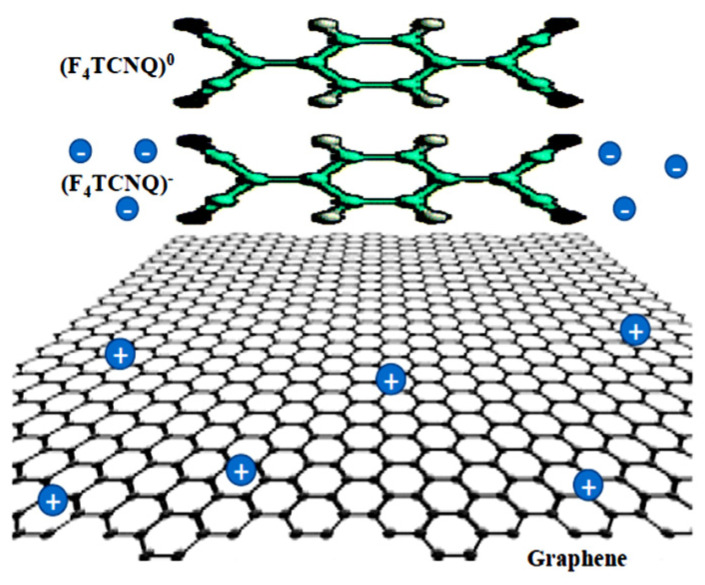
The structure of F_4_TCNQ molecules and e^−^ transferring from graphene to F_4_TCNQ.

The TCNQ derivative 2,3,5,6-tetrafluoro-tetracyanoquiondimethane (F_4_TCNQ) is a strong electron acceptor, widely used to improve the performance of graphene TCEs for various devices. For example, adsorbing F_4_TCNQ on graphene causes a significant electron transfer from graphene to F_4_TCNQ [178]. The F_4_TCNQ molecule is adsorbed at the bridge position, which has been reported as a stable adsorption site in [47,178,179]. There are systems consisting of a gate electrode, SiO_2_, graphene, and F_4_TCNQ, where the SiO_2_ and the gate electrode are approximated as continuum materials. The dielectric constant of the SiO_2_ is given an experimental value of 4.0 [180]. Removing the gate electric field causes the electric charge below the graphene layer to decrease, while the electric charge above the F_4_TCNQ layer increases due to a repulsive interaction between the charges in the graphene and F_4_TCNQ moieties. The amounts of the charge in the moieties without the gate electric field are close to each other: 3.5 × 10^−2^ (e^−^) for the graphene and 3.4 × 10^−2^ (e^−^) for the F_4_TCNQ [181]. Pinto and co-authors [11] showed that the hole concentration of epitaxial graphene (EG) could be controlled by the deposition of F_4_TCNQ. Thus, the F_4_TCNQ molecule is a p-type dopant for graphene. After 0.1 nM and 0.2 nM F_4_TCNQ deposition, the work function increased from 4.0 eV (pristine graphene) to 4.7 eV and to 5.3 eV, respectively. F_4_TCNQ has also been known to dope p-type carbon nanotube field-effect transistors, where both the channel resistance and contact resistance have been reduced by the layer deposition of F_4_TCNQ molecules [182]. Figure 10 shows the structure of F_4_TCNQ and the doping scheme of graphene with F_4_TCNQ. The molecule adopts an approximately planar geometry 3.1 A above the graphene sheet. These results are in agreement with synchrotron-based high resolution photoemission spectroscopy measurements, which show an increase in the work function with an increase in the thickness of F_4_TCNQ on the top of graphene [53].

**Figure 11:**
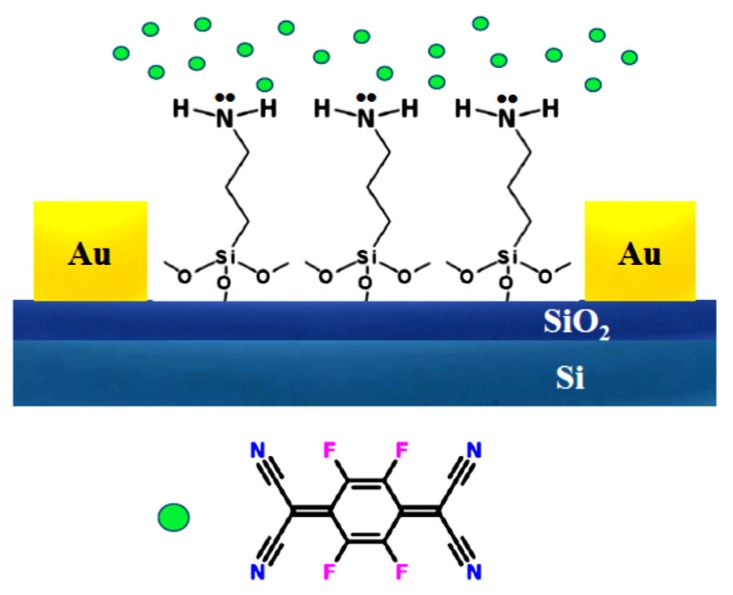
Schematic dual doping process of Au (50 nm) for bilayer graphene consisting of both n-doping by aminopropyltriethoxysilane self-assembled monolayers (NH_2_-SAMs) and modified SiO_2_/Si substrate (bottom).

Park and co-authors in [52] suggested that the inhomogeneous morphology of F_4_TCNQ thin film may induce fluctuations in the doping degree, thus causing the formation of disorder-induced electron–hole puddles (Figure 11).

In addition, the presence of four fluorine atoms enables F_4_TCNQ^n−^ (n = 0, 1, or 2) to engage in strong hydrogen bonding or other stabilizing non-covalent E∙∙∙F contacts (e.g., E = S, Se), which can promote intermolecular association and electronic interactions [183,184]. The strong p-doping indicates a strong coulombic interaction resulting from the formation of interface dipoles between the F_4_TCNQ molecules and graphene [185]. However, the stable doping is hampered by the degradation of F_4_TCNQ molecules under ambient conditions by oxygen and water vapor.

## 5. Metal–TCNQ Charge-Transfer Complexes as Electrode Materials

Most one-dimensional organic micro-/nanomaterials have attracted considerable interest over the past four decades due to their intriguing structural, optical, conducting, and switching properties. The metal micro-/nanocrystals of TCNQ, in the size range of 100–2000 nm, can also be attached to the surfaces of graphite, glassy carbon, gold, and platinum electrodes by the process of dry abrasion. For example, when the resulting surfaces are placed in aqueous solutions of group I cations, such as Na^+^, K^+^, Rb^+^, and Cs^+^, and the electrode potential is cycled, reversible phase transformations occur between TCNQ and its corresponding cation salts. The combination of optical microscopy, scanning electron microscopy, and X-ray diffractometry provides deep insight into the relationship between the electrochemistry and crystallography of these crystals [126]. In 2013, Ramanathan and co-authors [186] reported very interesting results with an organic semiconductor made from potassium tetracyanoquinodimethane (KTCNQ), with the possibility of charge transfer to textiles for flexible organic electronics. The authors demonstrated the stability of the material in the construction of new electronic switches and gas sensors. The ability to grow KTCNQ nanorod arrays in a radial symmetry directly on textiles as a versatile 3D microtemplate can be extended to the synthesis of various metal–organic charge-transfer complexes on different flexible substrates. This has a great potential for use in future flexible optical, sensing, and electrochemical catalytic systems.

Given that TCNQ is an extremely good electron acceptor, transition metals such as Cu, Ag, Mn, Fe, Co, Ni, Zn, and Cd have been chosen for the synthesis of various metal–TCNQ compounds. While each of these metal–organic charge-transfer complexes shows unique physicochemical properties, they have been primarily investigated for electronic applications [187,188]. The electrochemistry of polimeric TCNQ derivatives based on the transition metals Ag, Cu, Co, Ni, Zn, Mn, Cd, and Fe have had particular aspects of their electrochemical behavior and redox properties studied. For example, Ren and co-authors [189] investigated the morphology and electrical conductivity of AgTCNQ, CuTCNQ, Co[TCNQ]_2_(H_2_O)_2_, and TTF-TCNQ (for comparison) CT complexes in the form of micro-/nanowires [189]. The authors suggested that the morphologies of these complexes differed significantly from one another, depending mainly on the used conjugated molecules. The morphologies were also related to the electrochemical reaction routes [190]. Using SEM, it was shown that the electrocrystallization of cobalt TCNQ ARS occurred at a potential ranging from 0.1 to 0 V vs. Ag/AgCl. This potential range was optimal to obtain well-separated crystalline needles of Co[TCNQ]_2_(H_2_O)_2_. The study highlights the importance of the electrocrystallization approach in constructing and controlling the morphology and stoichiometry of Co[TCNQ]_2_-based materials. The authors also noted that the electrical conductivities of the electrochemically obtained CT complex micro-/nanowires were much lower than that of the bulk single crystals. In another work, they observed that the electrical conductivity of TTF-TCNQ nanowires was 0.013 S cm^−1^, while that of TTF-TCNQ bulk single crystals was 500–1000 S cm^−1^ [189,191]. Ren et al. [189] concluded that a tiny CT nanoelectrode was particularly effective for synthesizing organic, conductive micro-/nanowires. Zhao and co-authors demonstrated that the electrocrystallization of AgTCNQ occurred in the potential range of −0.1 to 0.3 V vs. Fc/Fc^+^ when Ag was deposited at electrode defect sites, followed by a reaction with TCNQ to produce 3-D structures ranging from nanotubes to nanowires [188]. At higher stoichiometric concentrations of Ag^+^ and more negative potentials (<−0.1 V vs. Fc/Fc^+^), extremely thin crystalline films could be obtained [187]. It has been shown that AgTCNQ and CuTCNQ can be used as new organic, electric, bistable films for ultrafast electric memories [188]. This is a material which can transit from a highly resistive state to a conductive state by an electric field. It can be used as a current switch, surge voltage protector, and voltage sensor. CuTCNQ has been investigated for about 40 years as a representative bistable material and the prototype of molecular electronics with great potential for applications in nanoelectronics [24,192,193,194,195]. Now, several theories regarding the switching effect in CuTCNQ thin films remain under discussion. The work in [192] noted that the reversible phase transition in the solid state is responsible for this resistive switching effect in CuTCNQ. An applied electrical field supports the transition of the reduced TCNQ form and oxidized Cu^+^ ions to their neutral state. Therefore, a phase transition in the polycrystalline AgTCNQ and CuTCNQ films is observed too. This transition is the basis for the optical switching effect in these salts. The CuTCNQ layer appears to be a suitable spacer, which possibly stabilizes the reversible switching by acting as a Cu ion buffer. In 1999, Heintz et al. obtained two phases of CuTCNQ: the high-conductivity (about 2.5 × 10^−1^ S cm^−1^) kinetically stable phase I and the low-conductivity (only 1.3 × 10^−5^ S cm^−1^) thermodynamically stable phase II, which could easily coexist in one system [196]. Nanowire, nanotube, and nanorod structures are the basis of phase I, while plate and lamellae microstructures are the basis of phase II. Phases I and II also differ in their bandgap values, which are 0.137 and 0.332 eV, respectively [24]. From this point of view, CuTCNQ micro-/nanostructures are very promising materials for applications in the construction of new electrode materials, sensors, and nanocones (a new morphology of 1D nanostructures). For example, CuTCNQ nanocones, due to their nanoscale tips, are potential organic materials in field-emission devices [197].The ratio of Cu:TCNQ is also of great importance for growth processes occurring in the these nanocones. The large area of CuTCNQ nanocone layers with various scale images was fabricated and investigated only in 2021 [24].

In 2018, Wu and co-authors obtained a stable CuTCNQ NA/CF (NA—nanorod array; CF—copper foam) three-dimensional glucose sensor based on direct electrochemical detection [198]. The sensor showed a response time within 3 s, a wide linear response in the range of 0.001–10.0 mM, and a low LOD of 10 nM (S/N = 3). Moreover, it also featured long-term stability and reproducibility, the highest selectivity, and the lowest LOD compared to other copper-containing sensors [199,200,201]. It has been successfully applied to the analysis of fresh human serum samples. It can therefore be concluded that CT complexes based on metal–TCNQ micro-/nanowires are a new area of research and open the gate to an unlimited number of new organic nanomaterials used as mediators in electroanalysis.

The electrosynthesis at the potential of −0.15 V vs. Ag/AgCl and characterization of semiconducting Ca(TCNQ)_2_ microsheets was described in [202]. The formation of different microsheets for parent TCNQ crystals and the needle crystals of Li, Na, K, Rb, and Cs cations was observed at this potential. This was proven by the research results of the SEM characterization of the morphology of 1D, 2D, or 3D Ca(TCNQ)_2_ microsheets. Ca^2+^ ions were very stable even when a bare glassy carbon electrode over the potential range of 0.45–0.10 V vs. Ag/AgCl in an aqueous solution of Ca(TCNQ)_2_ was used. Nafady and his team suggested that the observed progressive decrease in the peak current was the main evidence that a TCNQ/Ca(TCNQ)_2_ transformation was taking place. Voltametric parameters significantly depend on the Ca^2+^ concentration and scan rate; however, they do not depend on the ITO, GC, Pt or Au electrode materials.

In 2020, Hussain et al. reported the unique morphological, vibrational, and optical properties of organic charge-transfer complexes based on Pb(TCNQ)_2_ and its fluorinated derivative Pb(TCNQF_4_)_2_ [203]. The authors noted that both the Pb film and Pb(TCNQ)_2_ appeared to be rather poor catalysts. However, the catalytic activity of the Pb(TCNQF_4_)_2_ for [Fe(CN)_6_]^3−^ reduction was significantly better that of the Pb(TCNQ)_2_. Thus, these materials are good redox catalysts, with the fluorinated derivative demonstrating a better performance by two orders of magnitude than the non-fluorinated Pb(TCNQ)_2_.

Iron tetracyanoquinodimethane, Fe(TCNQ)_2_, is an example of a 3D electrode with a working potential range from +1.12 to +1.22 V vs. RHE [204]. Iron is a low-cost and naturally abundant transition metal, which has been widely used as an effective dopant to enhance oxygen evolution reaction (OER) activities [205,206]. Fe(TCNQ)_2_ possesses outstanding electric conductivity. In [204], Fe(TCNQ)_2_/CF, Hg/HgO, and a graphite plate were used as working, reference, and counter electrodes, respectively. Polarization curves were registered in a 1.0 M KOH electrolyte with a scan rate of 2 mV s^−1^. This low-cost Fe(TCNQ)_2_ material is a promising catalyst for the application of OER due to its good stability and high anodic current density of Fe(TCNQ)_2_/CF. CuTCNQ can also be used as an anode material for the photoelectrochemical oxidation of water [207].

Particular attention has been paid to the two-dimensional organic coordination compounds of 3d metal–TCNQ after Thompson’s group’s research in 1979 [208]. The authors described the magnetic and electric properties of Mn(TCNQ)_2_ and its hydrates and found that the Mn–Mn exchange interaction and electrical conductivity increased with a decreasing water content. In 1983, Endres described in detail the synthesis and properties of TCNQ ARS with simple and complex metal cations [209]. Much later, in 2018, Blanco-Rey et al. studied and described the formation of two-dimensional 3d transition metal–organic networks a Au(111) metal surface modified by organic TCNQ ligands [210]. These networks were magnetic anisotropy systems with exchange coupling. In 2020, the unique properties of TCNQ anion radical salts with [M(N-N)_2_]^2+^ cations (M = Mn, Fe, Co, Ni; N-N = phen, dips) were analyzed [211]. The authors considered the ability of the anion radical salts to create conductive materials, magnetically ordered structures, including ferro- and antiferromagnetic ones, spin ladders, and materials for the production of field-effect transistors and photo-diodes in the electronics of nanomaterials. In 2018, Liu and et al. developed a Mn(TCNQ)_2_ nanorod array with long-term stability in alkaline media [212]. As a 3D catalyst electrode in 1.0 M KOH, the Mn(TCNQ)_2_/CF exhibited a marvelous electrocatalytic performance with a low overpotential of 306 mV, as well as a long-term electrocatalytic stability. The present findings show its potential practical application for OER, like Fe(TCNQ)_2_.

Transition metal-based single atom catalysts supported by TCNQ monolayers have been suggested as interesting electrocatalysts for nitrogen [213], oxygen [214], and CO_2_ reduction reactions [215]. For example, a recent study conducted by Hassan et al. showed highly stable transition-metal homo-/hetero-diatomic catalysts based on Al, Be, B, and Si supported by a TCNQ monolayer with intriguing properties as potential candidates for the electrocatalytic improvement of CO_2_ conversion [216]. The hetero-diatomic 2D AlBe-TCNQ and BeSi-TCNQ could effectively capture and activate CO_2_ molecules with binding energies ranging from 0.09 to 2.35 eV, while significantly higher binding energies of 1.60, 0.70, and 1.98 eV were found for the homo-diatomic 2D Al_2_-TCNQ, B_2_-TCNQ, and Be_2_-TCNQ catalysts for CO_2_ adsorption. The authors found that the TCNQ-based catalysts could easily adsorb and activate CO_2_ due to their structural stability. The CO_2_ adsorption occurred through carbon and one oxygen atom, leading to an increase in the C-O bond length and greater charge accumulation in the adsorber, which was indicated by an increase in the cyan color intensity.

Lin et al. developed and described electroanalysis in a standard configuration of a three-electrode cell with a Pd microdisk working electrode on a Si wafer in CH_3_CN, with applied potentials between −100 and 120 mV vs. Ag/AgCl for the electrodeposition of CoTCNQ for NO_2_, an important industrial gas-sensing application [217]. The short fabrication time of the tested sensor, its good sensitivity, and its high selectivity at room temperature can be useful for developing sensors of a low power consumption as compared to metal oxide gas sensors [218].

In summary, TCNQ and its reduced form are ideal candidates for the synthesis of new METCNQ materials by electrochemical reduction. TCNQ is one of the strongest electron acceptors which can very easily create TCNQ^−^ and TCNQ^2−^. This process can be achieved using TCNQ-modified electrodes containing transition metal electrolytes in aqueous media. TCNQ^−^ and TCNQ^2−^ are created by electrocrystallization from solid–solid transformation in organic solvents because TCNQ and its derivatives dissolve easily in these solvents. As result, a sufficiently large quantity of high-quality single crystals based on TCNQ can be obtained. The size of MeTCNQ crystals depends on the TCNQ and Me^2+^ concentration and electrolysis time, especially in the case of nanowires. The density orientation and shape of M(TCNQ)_2_ crystals (M = Ni, Zn, Mn, Cd) depend on applied potential and the nature of the electrolyte anion and metal ion. It has also been emphasized that their final morphology is the result of competition between solute diffusion and growth anisotropy.

Future developments are expected in this research area through the use of fluorescent materials and other positively charged molecules, including amino acids. These biological species can react with TCNQ^−^ and TCNQ^2−^ anions, forming new materials with interesting physicochemical, biological, electrochemical, and optical properties. These properties constitute the great achievements of recent years, expanding the applicability of metal–TCNQ materials in electroanalytical areas. The major applications of metal–TCNQ compounds are summarized in Table 2.

The superhydrophobic surfaces of the micro-/nanocrystals of the metal–TCNQ system allows for their potential use in water-/moisture-resistant and contamination-free nanoelectronic devices. All their promising properties have provoked much contemporary research focusing on energy and data storage, optical and electrical media recording, electrochemistry, and nanotechnology, as well as sensors and catalysis [204,213,214,215,216,217,218]. The results presented in this part also provide new opportunities to use organic–metal–TCNQ semiconductors for reductive photocatalysis applications [203,219,220].

## 6. TCNQ Metal–Organic Frameworks as Promising Active Materials for Chemiresistive Sensors

Metal–organic frameworks (MOFs), also known as porous coordination polymers (PCPs) or porous coordination networks (PCNs), are architected through coordination bonds between inorganic–metal nodes and organic ligands [221]. One of the challenges in using MOFs as porous materials is making them optimally conductive for application as electronic components and electrode materials. The high internal surface areas of MOFs are suitable for gas sorption [222], electrochemical sensors [223], and Lewis acid catalysts [224,225]. MOFs built with a TCNQ as a linker exhibit an excellent selective uptake of O_2_ and NO [226] in comparison to N_2_, CO_2_, C_2_H_2,_ and Ar, as well as the separation of benzene from cyclohexane [227], due to guest–host charge-transfer interactions. Quite recently, it was discovered that the infiltration of TCNQ in an MOF through covalent interactions can dramatically improve electrical conductivity and redox activity [56,228,229]. For example, it is also known that infiltrating a high-surface-area microporous material, HKUST-1, with TCNQ induces an increase in the electric conductivity of the MOF by over seven orders of magnitude (from <10^−8^ S cm^−1^ to ~0.1 S cm^−1^) [229]. In this case, neither the MOF nor TCNQ is conductive in its pure state.

MOFs are complex materials that exhibit a range of structure-directing phenomena, energetics, electronic structures, and length scales that are relevant to how they interact with guest molecules. Guest@MOF materials based on TCNQ have so-called “emergent” properties (electronic conductivity and efficient energy transfer), and the interaction between the guest and framework leads to a new unique class of materials based on MOFs with a variety of potential applications [230]. In 2015, Allendorf and co-authors described TCNQ@HKUST-1 as an electronically conductive Guest@MOF material [231]. They deposited thin HKUST-1 films on SiO_2_ substrates patterned with Pt electrodes and then infiltrated them with TCNQ by immersion in a saturated methanolic or dichloromethane solution of TCNQ at room temperature. The authors showed that the current–voltage characteristics were linear over a large potential range, and the current scaled linearly with the channel length. The conductivity of the MOF-TCNQ@HKUST-1 films was at least 10^7^ higher than that of the infiltrated MOF. The authors suggested the possibilities of creating electronically conductive MOFs based on TCNQ. Thus, MOF-TCNQ systems hold potential for use as electronic and optic structures with promising properties.

**Figure 12:**
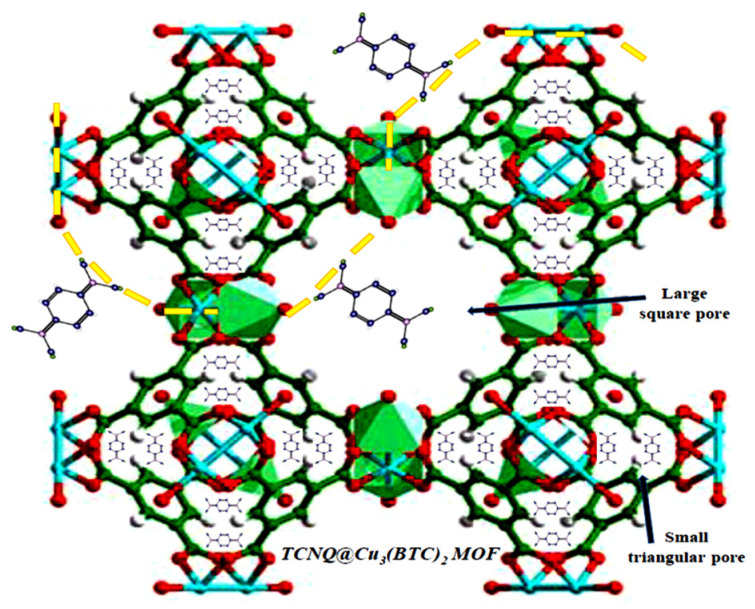
A schematic representation of the formation of a continuous chain through the MOF crystal in a conductive TCNQ@Cu_3_(BTC)_2_ MOF (the yellow dashed line indicates the pathway for charge conduction).

In 2021, Kim and co-authors described a new Guest@MOF system, where the MOF was Cu_3_(BTC)_2_ (BTC—benzene-1,3,5-tricarboxylate) [232]. Molecules such as H_2_O adsorb open metal sites in Cu_3_(BTC)_2_-MOF pocket, while small nonpolar and quadrupolar molecules such as CO_2_ or CH_4_ can be perfectly adsorbed by the tetrahedron side pockets, which may be significantly less impacted by the TCNQ guests [233,234]. The authors developed a synthetic pathway for novel TCNQ@MOF hybrid systems that could be useful for many MOF-based applications such as gas separations and electrochemical sensors performed under humid conditions and for the development of TCNQ-MOF/CO_2_ sensors. Earlier, in 2017, the TCNQ@Cu_3_(BTC)_2_ complex was also described [235]. In this case, TCNQ molecules are coordinated in a bridging geometry at positions 7 and 8 through C≡N groups to two copper paddlewheel secondary building units (SBUs) in Cu_3_(BTC)_2_ pores (Figure 12). The presence of one TCNQ molecule per pore allows the formation of a continuous chain through the MOF crystal to be observed. Cu(II) dimers become bridged by the TCNQ molecules, forming an electric conduction pathway and increasing from <10^−9^ to ~0.1 S m^−1^. This result makes the TCNQ@Cu_3_(BTC)_2_-MOF promising for further exploration as an electrode material in electrochemical sensing platforms [235,236].

The conclusion that TCNQ@Cu_3_(BTC)_2_ metal–organic frameworks could be used as electrode materials was made in the same year by Bhardwaj and co-authors [237]. They studied and described an anti-PSA-conjugated TCNQ@Cu_3_(BTC)_2_ immunosensor for the prostate cancer antigen (PSA) in the range of 0.1–100 ng/mL (LOD = 0.06 ng/mL). This biosensor was made by connecting the conducting platform based on TCNQ with antibodies. It could even work in the presence of other active substances including proteins.

In 2022, Lüder and co-authors described conductive MOF based on two different organic ligands, 2,3,6,7,10,11-hexahydroxytriphenylene (HHTP) and 7,7′,8,8′-tetracyanoquinodimethane. These architectures had a good electrical conductivity of 0.033 S cm^−1^ and a chemiresistive response toward ambient changes [68]. Temperature-dependent measurements demonstrated that the electrical conductivity of Cu-HHTP-TCNQ around room temperature could be described by a thermally activated process with an activation energy of about 0.142 eV [238]. The authors showed that the hybrid Cu-HHTP-TCNQ-MOF architecture was a promising active material for chemiresistive sensors, without the need for high crystallinity, and for future work toward the development of HHTP-TCNQ-based chemiresistive sensors.

In another work, Co(TCNQ)_2_-MOF was described as a high-efficiency catalyst for an oxygen evolution reaction [64]. The investigation of the electrocatalytic Co(TCNQ)_2_/Co OER performance depended on a typical three-electrode system. Co(TCNQ)_2_/Co was used as the working electrode, and a graphite plate and Hg/HgO were the counter and reference electrodes, respectively. Moreover, the system Co(TCNQ)_2_/Co(TCNQ)_2_^+^Co/Co foil was also very electroactive and demanded an overpotential of 339 mV for 15 mA cm^−2^. It was also found that the Co(TCNQ)_2_/Co possessed a small R_ct_ value (2.3 Ohm), which suggested a high charge-transfer rate and rapid catalytic kinetics [239,240]. The strong intermolecular charge transfer reduced the optical band gap down to 1.5 eV of divalent TCNQ and enhanced the electrical conduction, which allowed the Co(TCNQ)_2_-MOF to also be utilized for resistive gas- and photo-sensing [241].

In 2023, an Ab/TCNQ-NiBTC/SPE (Ab—antibody; SPE—screen-printed electrode) immunosensor was developed for the determination of von Willebrand factor (VWF) antibodies [242]. The sensor was used to investigate the release of VWF from H_2_O_2_-induced oxidative-injured human umbilical vein endothelial cells. The authors reported that the electrochemical properties of NiBTC and the linker of MOFs were enhanced when combined with TCNQ.

Recently (in 2024), Angel and co-workers obtained conductive metal–organic frameworks, TCNQ@M-MOFs-74 (M = Mg, Mn, Cu, and Zn), exhibiting a striking correlation between their bulk conductivities and open d-shell of Cu and Mn, arising from the TCNQ p-doping of the MOFs [243]. The presence of conjugation in the guest molecules is critical in completing a molecular network necessary for inducing conductivity in TCNQ@M-MOF-74, as was earlier observed for TCNQ@Cu_3_(BTC)_2_ [229,235]. This work was a big and important step toward understanding how alternative charge-transport pathways may help access conductive behavior in insulating inorganic parent materials.

In 2024, Mukherjee et al. fabricated and described the 3D nanoarray architecture of metal–organic frameworks based on an Fe(TCNQ)_2_ complex on copper foam (CF) as an efficient electrocatalyst [244]. It had a high ammonia yield rate of 11,351.6 mg h^−1^ cm^−2^ and a faradaic efficiency (FE) of 85.2% on the electrode, with a catalytic stability time of 2 days at −1.1 V vs. RHE. The findings show great potential for the exploration of other transition metal–TCNQ-based electrocatalysts for the electrochemical reduction reaction of nitrate (NO_3_RR) to ammonia. This compound is widely produced on a global scale due to its importance as an intermediate form of hydrogen energy storage.

**Figure 13:**
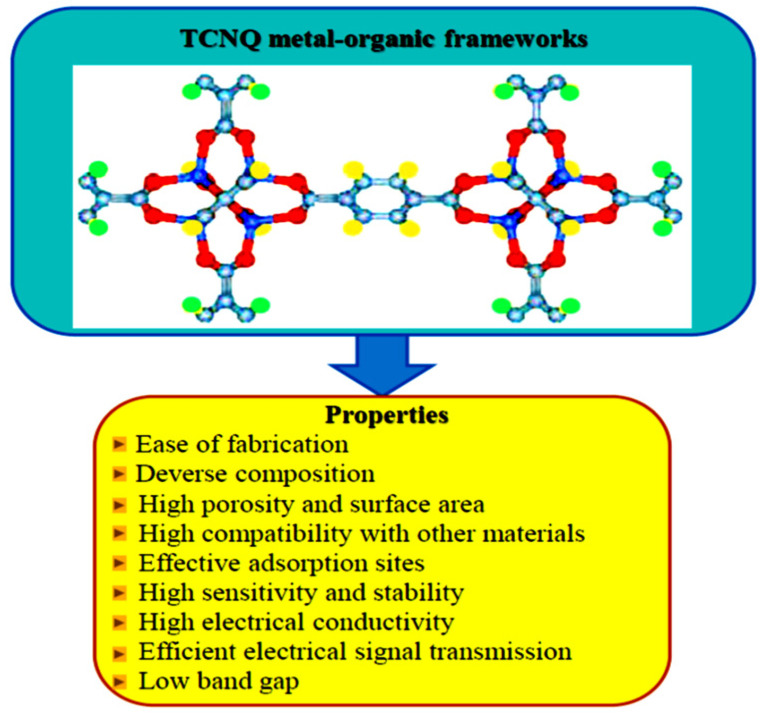
Schematic illustration of properties of TCNQ-MOFs for chemiresistive sensors.

The above results indicate that the most extensively studied example is copper-based TCNQ-MOF. Only recently have electrically conductive MOFs been obtained by doping with a redox-active TCNQ molecule. The electronic gap is reduced by doping, and charge-transfer TCNQ@MOFs should become more conductive. Thin films of doped MOFs synthesize directly on the electrodes. The coordination polymerization leads to various metal–organic frameworks with unique physical properties and chemical functionalities (Figure 13). Such interesting properties as high selectivity and sensitivity are the basis for the use of these chemical sensors in a wide range of applications, for example, in medical diagnosis, food quality control, and in toxic gas monitoring. MOFs with record-breaking, large surface areas are perfect materials in the sensing of different analytes.

Electrochemical sensors based on TCNQ-MOFs exhibit unique and very promising sensing properties. These systems, with a low band gap or high electrical conductivity, can effectively transfer electrical signals. Many of their composite materials have been recognized as next-generation electrodes for the development of efficient supercapacitors. Now, it is possible to design and develop the shape, size, porosity, phase, and conductivity of TCNQ@MOF materials by selecting the proper organic or inorganic constituents of the MOF precursors and controlling heat treatment conditions. Due to the significantly stranger interactions of TCNQ with MOFs, the new, stable TCNQ@MOF architectures are not subject to structural degradation, even in water, compared to those of water molecules competing for the same framework binding sites. The introduction of organic molecules, particularly TCNQ, into MOFs marks a turning point in the development of MOF chemistry and will continue to play a key role in their future development to obtain novel structures, properties, and applications, specifically as sensitive platforms for building electrochemical sensors.

In addition to MOFs, covalent organic frameworks (COFs) have been extensively investigated during the past two decades. This is a class of crystalline organic polymers with highly ordered structures [245]. π-electronic skeleton-based COFs with highly ordered porous architectures can be used as a material platform for structural and functional designs and as an electrode material with good stability at high potentials [246,247,248,249]. The conductivity of COFs is limited by disordered regions and particle boundaries. The introduction of redox groups into COFs leads to good electrochemical performance and the emergence of new COF-based materials for electrochemical sensing applications [247,250,251,252]. The COF films also show high electrical conductivity (c.a. 3.4 S m^−1^) after being doped with TCNQ; this is a new record for 3D COF materials [253]. A slight increase in conductivity is observed with an increasing channel length (the distance between electrodes).

In 2014, three thermally stable TTF-COFs were obtained [254,255,256]. For example, in the work in [254], the TTF-COF film was doped with nonvolatile TCNQ by immersing it in a CH_2_Cl_2_ solution. The resulting TTF-TCNQ-COFs were stable, with tunable electrical conductivity, and remained unchanged when exposed to air over 24 h.

In 2018, Li and co-authors prepared a new class of conductive, microporous TCNQ-CTFs (CTF—covalent triazine-based framework) from the trimerization of TCNQ in molten ZnCl_2_ followed by further rearrangement at higher temperatures (from 400 to 900 °C) [257]. In this case, the resulting products were denoted as TCNQ_CTF-400 and TCNQ-CTF-900. The authors observed that the CV curves showed impressive redox peaks at a low scan rate of 2 mV s^−1^, which was assigned to the nitrogen atom in the frameworks of TCNQ-CTF [258]. In another work, TCNQ-CTF-800 with a large surface area and a longer discharge was described [259]. The TCNQ covalent triazine-based frameworks could be used as supercapacitors. These materials have a large surface area too, as well as a high nitrogen doping concentration for different applications.

In 2020, a new COF with a long-term, stable, high electrical conductivity (3.67 S m^−1^) was obtained by using a strong organic acceptor, F_4_TCNQ [260]. Such stability is important to exploit high conductivities by doping permanently and to use a F_4_TCNQ-COF for various optoelectronic applications.

In conclusion, when TCNQ-COFs are redox-active and also change color due to the electrochemical redox reaction, they can be used as functional layers in electrochromic (EC) devices. Their ultrahigh surface area, lightweight and precisely controlled pore and atom distribution, high stability in a wide range of solvents and conditions, and high electrical conductivity show that electroactive TCNQ-COFs are an attractive alternative to traditional materials in many electronic applications. TCNQ-based COFs can also be good candidates in the disciplines of electrochemical energy storage (EES) and electrochemical energy conversion (EEC).

More recently, a family of hybrid materials, MOF@COFs, has emerged as particularly appealing for gas separation and sensing, catalysis, drug delivery, and plant disease diagnosis [251,259,261]. MOF@COF hybrids combine the unique characteristics of both MOF and COF components and exhibit distinctive properties, including high porosity and a large surface area. MOF@COF-TCNQ also shows excellent energy storage properties [259].

## 7. TCNQ and Its Derivatives as Cathode Materials in Batteries

Supercapacitors and batteries are two of the main ways of achieving energy storage. With the development of electronic devices, the power density and energy density of batteries must be higher. Organic electrode materials, which use redox-active organic compounds as active materials, are attracting attention as an alternative to conventional inorganic electrodes [262,263]. The batteries using organic electrodes and an aqueous electrolyte are promising because they offer safety, stability, and environmental friendliness [264,265,266]. The application of organic conductors in battery electrodes is one of the solutions to considerably improve the electrical conductivity of small organic molecules. The structural factors of the working electrode, including the surface area, morphology, spatial distribution of the active material, and connectivity with the current collector, greatly affect the overall performance of battery systems and are becoming increasingly important for new types of energy storage systems such as batteries. TCNQ with high conductivity is identified as an optimized cyano-organic positive electrode material. As a typical structure with strong polarity, the cyan group (C≡N) can be easily reduced and regenerated by further oxidation [267,268,269]. The redox behavior and electric energy levels of cyan groups can be effectively manipulated by changing their molecular structures [270]. TCNQ and its derivatives possess the highest redox potential among n-type redox-active organic compounds (2.4–3.2 V vs. Li^+^/Li); therefore, TCNQ ARS and CTCs can be synthesized with more than 90 wt% of active material. Electrode-level-specific energy can be achieved even with a small amount of conductive agent (less than 20 wt%). Enhanced cycle stability has been achieved for organic electrodes with TCNQ [271,272,273,274].

The invention of the first commercial lithium ion batteries (LIBs) in the 1990s marked a turning point in the technological revolution. The main problem in this field of research is that effective contact between the electrolyte and the electrode-active material is difficult to achieve, which reduces the energy density and battery performance. To solve this issue, researchers have looked at the composition of electrodes. The active material in an electrode can help lose or gain electrons through redox reactions and enables charge transfers between the electrode and the electrolyte. After almost three decades, in 2019, Lee et al. reported an organic CTC as cathodes in a lithium ion battery to enhance its electrical conductivity [273]. This was the reason why a novel electrical conductivity-relay system was proposed in 2020, which imparts electrical conductivity to organic small molecular electrodes without any conductive additive throughout the charge/discharge cycles [70]. Aqueous batteries have been regarded as a potential alternative to LIBs for large-scale energy storage, considering safety, productivity, and economic and ecological aspects. Fujihara and co-authors [70] developed a mixed molecular crystal couple (MCC) of TTF and TCNQ as a cathode without any conductive additive. Electrochemical tests with a three- electrode cell (from -0.6 V to +0.6 V) using a 1 M NaBr aqueous electrolyte were described. TTF-TCNQ MCC, activated carbon pressed on a Au mesh current collector, and Ag/AgCl were used as the cathode working electrode, the counter electrode, and the reference electrode, respectively. An overshooting potential was observed, at 0–10 mAh×g^–1^ and 0.1–0.2 V, with the CT complex cathode. Such behavior has often been observed in cathode materials for LIBs [274,275].

Common factors such as the degradation (aging) of an electrode or electrolyte greatly affects the life cycle and cycle stability of all rechargeable batteries, including conventional lithium ion batteries, during electrochemical reactions [276]. The low power density and volumetric energy value of organic rechargeable batteries means that these batteries are disadvantageous [277]. However, redox-active organic materials are considered environmentally friendly and can even be obtained from natural products with a minimal environmental impact [267,278].

Over the past few years, aqueous Li (Na, K) ion batteries have attracted extensive attention due to their low toxicity and nature of high safety. However, Li (Na, K) intercalation compounds generally exhibit a low capacity (less than 150 mAh×g^−1^) in an aqueous electrolyte [279]. It is well known that CuTCNQ, as a model electrode material, can reversibly store Li^+^/Na^+^/K^+^ ions under lower working voltages [280,281,282,283]. The electrochemical activity of the Cu^2+^ cation and TCNQ^−^ anion means that redox CuTCNQ electrode material can take part in multi-electron transfer reactions.

For example, in a 2019 study by Meng and co-authors, a self-supported CuTCNQ film was fabricated as an electrode for LIBs [280]. It was found that the first discharge and charge-specific capacities of the self-supported CuTCNQ electrode were 373.4 and 219.4 mAh×g^−1^, respectively. After 500 cycles, its reversible specific capacity reached 280.9 mAh×g^−1^ at a current density of 100 mA×g^−1^. This result was also obtained in [284]. These data reveal that a high capacity is ascribed to the multiple-electron redox conversion of both metal ions and ligands, as well as the reversible insertion and desertion of Li^+^ ions into the benzene rings of ligands. The conductivity of CuTCNQ film on Cu foil is 0.305 S cm^−1^, which confirms that CuTCNQ exhibits a high electrical conductivity [280,285]. Thus, Meng et al. concluded that CuTCNQ/Cu can be used directly as a self-supported electrode and as a promising candidate to design Li-based batteries.

In another work, in 2020, Yin and co-authors described a lithophilic CuTCNQ–metal–organic framework thin layer (CuTCNQ-MOF TL) with a high conductivity and large surface area [286]. The CuTCNQ-MOF layer, with its polar surfaces and porous structure, was found to have a very high affinity with the organic electrolyte and a strong ability to adsorb Li ions from the electrolyte. The electrochemical tests of the Li/CuTCNQ-MOF electrode revealed the very stable overpotential of the system, around 13.1 mV. Thus, the CuTCNQ-MOF layer could effectively inhibit the growth of Li dendrites and is a promising candidate for the design of high-performance metal-based batteries. Interestingly, in 2022, Wang et al. synthesized copper-tetracyanoquinodimethane, a charge-transfer metal–organic compound, and lithium-/sodium-based dual-ion batteries (DIBs) [287]. They were constructed by coupling the CuTCNQ anode with the graphite cathode. The graphite-based electrode was composed of graphite, acetylene black, and PVDF by casting the slurry onto an Al foil. In this work, lithium metal was used as the counter electrode, polypropylene membrane as the separator, and 1 M LiPF_6_ in ethylene carbonate/dimethyl carbonate/ethyl methyl carbonate (EC/DMC/EMC 1:1 vol%) as the electrolyte. As found, the DIBs provided a high–discharge voltage of 4.27 V and reversible capacities of 76, 64.7, 55.4, 45.3, 34.7, and 34.5 mAh×g^−1^_,_ with increasing current densities from 0.1, 0.3, 0.5, 1, and 3 to 5 A×g^−1^, respectively. The results demonstrated both the excellent rate capability and good reversibility of the DIBs. H. Wang and co-authors suggested that CuTCNQ could be used as an anode material to develop a new dual-ion batteries with the potential advantages of a higher working voltage, good safety, environmental friendliness, and low costs.

**Figure 14:**
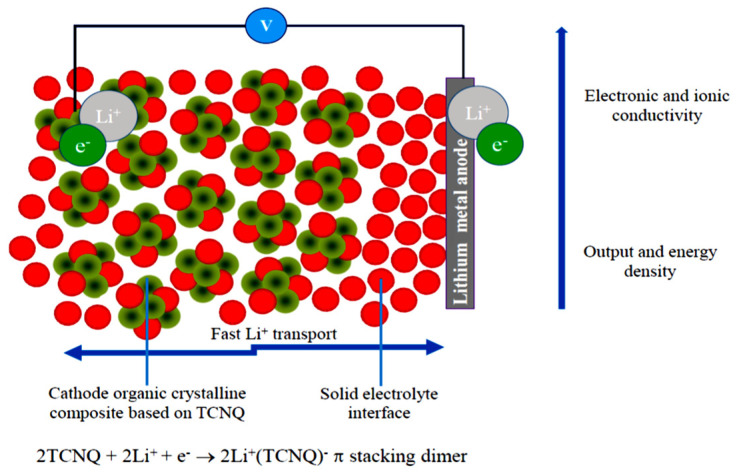
A schematic illustration of the construction of stable solid-state Li/TCNQ batteries.

In 2022, a high-voltage (3.4 V) solid-state organic lithium battery based on an F_4_TCNQ cathode in 1 M LiPF_6_ EC/DMC electrolyte with a capacity of 80 mAh×g^−1^ at a current density of 10 mA×g^−1^ was reported [288]. Geng and co-authors found that the long-life Ta-doped F_4_TCNQ/LLZTO-PEO/Li battery (LLZTO—Li_7_La_3_Zr_2_O_12_; PEO—poly(ethylene oxide)) had a relatively high redox potential of 3.0 V vs. Li^+^/Li. This is higher than the average redox potential of most organic cathode materials [289,290,291,292,293]. The authors concluded that the new, highly stable Li/F_4_TCNQ energy sources, with an electrochemical window from 2 to 4 V to 2–3.6 V and high resistance (over 25 kW), could be used to build organic solid-state lithium batteries with high voltages. Figure 14 schematically displays the construction of new, highly stable organic solid-state lithium batteries based on TCNQ or its derivatives.

Among aqueous batteries, Zn-based batteries have shown their considerable potential in practical applications: Zn metal has high theoretical capacity (820 mAh×g^−1^), low toxicity, high safety, and low cost. In 2020, Chola and Nagarale described TCNQ as a cathode material for an aqueous zinc battery with zinc plates as an anode in 1 M ZnSO_4_ [294]. Generally, the metallic zine anode is one of the safest electrode materials in aqueous batteries due to its good compatibility with water, high zinc abundance, environmental friendliness, and low redox potential (0.76 V vs. SHE) [295,296]. The cyclic voltammograms recorded in 1 M ZnSO_4_ aqueous solution showed an oxidation peak at 1.4 V whereas a reduction peak at 0.65 V [294]. This is due to the coordination or decoordination of Zn^2+^ ions with TCNQ molecules [294]. The nature of the peaks indicated that there are two very stable states of TCNQ, the reduced one and the Zn-coordinated complex. The results showed the effectiveness of using TCNQ as a cathode material for rechargeable Zn-ion batteries. A small amount of TCNQ inside cyanuric chloride-pyrene (CCP) or organic porous polymers could increase the stability of the TCNQ-based cathode.

Cyano-organic molecules are more promising materials for positive electrodes in zinc and aluminum batteries. Specifically, TCNQ provides a higher capacity than graphite positive electrodes (c.a. 60 mAh×g^−1^). The electron-deficient cyan groups in organic molecules can reversibly coordinate/dissociate with positively charged Al-complex ions [297,298]. In 2021, Zhong et al. obtained and described TCNQ cathode material for the construction of a battery with a Al^3+^ cation in an aqueous Na_2_SO_4_/Zn SO_4_ electrolyte [69]. In this aqueous electrolyte, the TCNQ cathode with a polypyrrole conductive coating layer (TCNQ@PPy) exhibited a perfect capacity of 245.8 mAh×g^−1^ at 0.5 A×g^−1^ and a life cycle of 1000 rounds at 3 A×g^−1^. The energy efficiency of the TCNQ@PPy-Zn battery was 75.4% at 0.5 A×g^−1^. According to [69], the calculated value of energy efficiency of the TCNQ@PPy-Zn battery was 75.4% at 0.5 A g^−1^. The TCNQ electrode had a very good reversibility (the coulombic efficiency ≈ 100%). The results showed that TCNQ was a good candidate for cathodes in aqueous batteries when adding highly valent cations. The addition of Al^3+^ improved the feasibility and reversibility of the insertion/extraction process of multiple cations, such as Na^+^, Zn^2+^, and Al^3+^, and also suppressed the dissolution of TCNQ.

In 2023, Wang et al. developed a microstructured, single-moiety, organic electrode material in aqueous zinc ion batteries (AZIBs) with initial capacities of TTF-TCNQ, TTF, and TCNQ of 153.4, 65.7, and 142.8 mAh×g^−1^ at 1A×g^−1^, respectively, at low temperatures [299]. Owing to its higher electrical conductivity, smaller charge transfer resistance, and lower activation energy, the TTF-TCNQ showed a superior rate and cycling performance, with a capacity retention of 80.9% and 74.2% after 270 and 500 cycles, respectively. This proves that TTF-TCNQ has an excellent zinc storage capacity and can be used as a new organic electrode material in AZIBs at low temperatures.

Also in 2023, Shao and his team developed a redox-active TCNQ-rGO (rGO—reduced graphene oxide) conductive network for aqueous ammonium ion batteries and investigated the electrochemical NH_4_^+^ storage–quinone redox mechanism of TCNQ-rGO [300]. The most vulnerable TCNQ bond to NH_4_^+^ transfer is the C=C_wing_ bond. The interaction between TCNQ and NH_4_^+^ is confined to C=C(CN)_2wing_, as determined by Raman [301]. The authors concluded that the obtained results provided strong evidence for the successful fabrication of TCNQ-rGO with an integrated 3D conductive network, favoring the electrochemical storage of NH_4_^+^_._

In another work, Wang et al. found that as the substituents of fluoride atoms as an electron-absorbing group on TCNQ molecules increased, their stability in aqueous zinc-ion batteries decreased [302]. Fluoride was selected for substituted and conductive groups to improve the average operating voltage and increase the redox potential of the organic molecule [303,304]. 

The TCNQ energy gap is 2.58 eV that is lower than that of most other organic electrode materials [305,306,307]. It is no wonder that TCNQ is of interest to many electrochemists. Standard electrode production methods are limited to a few conductive polymers such as polypyrrole, polythiophene (PTh) or polyaniline (PANI) [308,309].

The new TCNQ/FTCNQ/F_4_TCNQ working electrodes, with redox potentials of 1.02 V, 1.20 V, and 1.40 V, respectively, were paired with zinc foil as the counter electrode, using a 3 M ZnCl_2_ aqueous solution. It turned out that the TCNQ-based electrode material was also suitable for low-temperature application. The authors found that after 50 cycles, the charge and discharge platforms of the FTCNQ and F_4_TCNQ materials disappeared, while the discharge platform of the TCNQ decreased. This indicates that the active substances of TCNQ, FTCNQ, and F_4_TCNQ dissolve quickly.

In summary, metal–organic compounds with redox-active metal cations and organic TCNQ anions can open up new possibilities for designing and preparing potential cathode materials for high-rate and long-life batteries with a high energy density (Table 3). The above data are among the best results reported for the high-potential cathodes of metal-ion batteries, guiding future research and the possible commercialization of high-capacity batteries.

## 8. Potential Limitations of TCNQ in Specific Field

TCNQ is insoluble in water [310]. This property allows chemically modified TCNQ electrodes to be placed in aqueous electrolyte media with minimal dissolution. Reduction processes occurring on TCNQ-modified glass electrodes in aqueous solutions of transition metals also lead to the deposition of metal–TCNQ coordination polymers [311,312]. TCNQ has a theoretically high specific capacity, but sometimes, when it is used as a cathode material in batteries, it suffers from fast performance decay because of material dissolution [69].

This compound unexpectedly exhibits extremely low fluorescence with a quantum yield (QY) of ca. 0.1 in nonpolar solvents [313]. In this case, the fluorescence QY decreases with an increasing solvent polarity. Therefore, this property indicates that TCNQ can be used as a sensitive indicator for the detection of a change in the local polarity in materials.

## 9. Conclusions and Perspectives

In this review article, we have presented the historical context of electrodes based on TCNQ and its derivatives from their early development and have outlined theoretical and experimental aspects of the electroanalytical applications of these compounds. We have presented some of the most interesting applications of TCNQ and its derivatives in electrochemistry as potential electrochemically stable biosensors, electrode mediators for different electrochemical investigations, and cathode materials in batteries.

A strong 7,7′,8,8′-tetracyanoquinodimethane electron acceptor containing in its structure four electron-accepting cyan groups (-CN) has a better intrinsic electric conductivity, effective surface, and energy gap (2.58 eV). This value is lower than that of most other organic electrodes [305,306,307]. These unique properties make TCNQ one of the most widely used materials in electronics and sensing. The materials that perform charge/discharge without the addition of many additives are limited to a few conductive polymers such as polyaniline (PANI), polythiophene (PTh), and polypyrrole [308,309]. The exploration of simple methods, other than polymerization, which make small organic molecular materials electrically conductive is crucial for advancing research on organic electrode materials.

As shown in the sections above, the introduction of TCNQ mediators increases both the response of chemical biosensors to substrates and the sensitivity of electrochemical determinations. The resulting lower activation energy suggests that TCNQ films can potentially induce the mild surface transfer doping of graphene and carbon nanotubes, leading to CNTs-based chemical and biological sensors with high sensitivity, in particular, for the analysis of analytes with a low molecular mass, such as glucose [21,26,27,28,95,103,105,116,117,120,121,122,123,124,125,127,128,138,140,155,156,157], enzymes [94,109,115,126,133,134,138,142], heavy metals [136,137], carbamate drugs [145], pesticides [146,147], nucleic acids, high-molecular-mass analytes [107,108,109], and various proteins [110,111,112,113,135].

The mixed ionic crystals of TCNQ (acceptor) and TTF (donor) display interesting electrical conductivity properties and show some promise of affording superconductivity at high temperatures. Therefore, electrochemically stable TTF-TCNQ has recently emerged as a key constituent for new applications as a redox-fluorescent switcher and, in bioanalysis, to optimize enzymatic electrode architecture. Its potential applications are also expected in organic photoelectric and electrochemical sensing [28,32,90,91,92,93,94,95,96]. In redox-active (bio)sensors, the organic salt TTF-TCNQ is coupled to the metabolic turnover of the substrate by an enzyme and acts as an electron-transfer catalyst.

The TCNQ derivative F_4_TCNQ possesses an even higher electron-accepting character than TCNQ, with an exceptionally high potential value of 5.2 eV [182]. Therefore, it is often used as a p-type dopant to enhance hole conductivity in sensing devices. It is expected to be an effective acceptor on electrode surfaces, particularly as a dopant of graphene [11,47,52,53,178,179,181].

Layer-by-layer molecular doping processes on graphene can be used to create electrochemically stable graphene-/TCNQ-stacked films for polymer solar cell anodes. This paves the way for next-generation flexible devices requiring high conductivity and transparency [46,144,170,171,172,177,274].

Metal–TCNQ micro-/nanocrystals may also be attached to carbon surfaces, graphene, gold, and platinum electrodes [6,127,189,190]. TCNQ materials allow the development of optoelectronic switchers, gas sensors, and organic, conductive micro-/nanowires [186,188,189,191,193,197]. Biosensors based on Me-TCNQ possess long-term stability and reproducibility, the highest selectivity, and the lowest LOD compared to other sensors [187,188,189,190,199,200,201,202,211]. Three-dimensional electrodes based on Me-TCNQ may open up new applications in oxygen evolution reactions [204,205,206,207,212], while two-dimensional Me-TCNQ catalysts are ideal candidates for gas sensing [213,214,215,216,217,218].

Electrochemically stable metal–organic frameworks based on TCNQ and its derivatives can exhibit extremely high porosity, exceptionally high surface areas, high electrical conductivity, high modularity, tunable pore sizes, and diverse functionality. They also offer great potential as revolutionary catalysts, drug carrier systems, elements of gas storage, sensors, smart materials, separation agents, and chemical sensors working under humid conditions [222,226,229,244,253,254,259,260]. Such diverse properties often result not only from the selected TCNQ molecules and TCNQ-based organic ligand building blocks but also from their distribution within the framework. The TCNQ-based organic ligands add flexibility and diversity to the chemical structures and functions of MOFs. With the highly tailorable nature and precisely engineered pore structures of TCNQ-MOFs, highly porous metal–organic frameworks based on TCNQ can facilitate the development of next-generation high-performance materials through state-of-the-art chemical design [64,65,68,227,228,229,230,235,241,243].

TCNQ and its derivatives are new, organic, high-voltage, high-capacity, and promising materials for practical applications in battery technologies, showing tremendous progress in recent years [70,279,299,300,302]. Their interesting redox behavior consisting of a single two-electron reduction/oxidation wave leads to a single charge/discharge plateau associated with a good charge storage capacity. TCNQ electrode materials have been applied in a wide variety of energy storage devices, including non-aqueous Li^+^-, Na^+^-, and K^+^-ion, dual-ion, multivalent metal, aqueous, all-solid-state, and redox-flow batteries [279,286,287,288,294,297,298,301]. Organic structures based on TCNQ and its derivatives have also been extensively investigated as interesting active electrode materials for rechargeable batteries due to their low cost, abundance, environmental friendliness, and high sustainability. Thus, they can be seen as a future alternative for battery technologies [314].

In summary, the above presented materials based on 7,7′,8,8′-tetracyanoquinodimethane show promising characteristics for its application in transparent conductive electrodes in organic sensing devices. They are particularly useful for the development of a simple, sensitive, accurate, microvolume, and low-cost analytical approach, which is suitable for rapid field tests and effective as an alternative to existing methods. It are characterized by properties such as a large area, extremely high flexibility, smooth surface roughness, lowered electrode potential, extremely low sheet resistance, and good transparency. The novel types of TCNQ-mediated systems have resulted in the development of numerous amperometric biosensors and biofuel cells for investigations by electrochemical methods. Biosensors based on TCNQ can detect compounds present in the environment, chemical processes, food, and the human body at low costs compared to traditional analytical techniques. The use of TCNQ in electrochemical biosensors lowers the limit of detection to unparalleled levels, opening the door to new biosensing applications. This may increase the speed and flexibility of assays, as well as enabling multi-target analyses, automation, and cost reduction in electroanalytical investigations. Thus, systems based on electrochemically active TCNQ and its derivatives with unique and desirable properties expand the candidates for organic electrode materials with high energy to various pairs and mixing ratios of small molecules. The examples reported to date are all commercially available.

We hope that the data and conclusions presented in this review will provide readers with a clear understanding of TCNQ electrochemistry and, more importantly, help to find good compromises for the preparation of new, useful electrode mediators for the rational design of multifunctional TCNQ and its derivatives. We believe that this work will provide further insight into the fabrication and applications of TCNQ-based electrode materials in electroanalysis and many energy-storage devices.

## Figures and Tables

**Table 1:** TCNQ and TTF-TCNQ as mediators for different biosensors.

Biosensor Composition	Substrate	Operating Potential/V vs. Ag/AgCl	Buffer/pH	LR/mM	LOD/μM	Ref.
Pt/TTF-TCNQ/SHE/GOx	Glucose	−0.2 to 0.3 (vs. SCE)	PhB/pH 7.4	0.2–100	1.4	[141]
PVC/KB-TCNQ/GOx/ polytetrafluorethylene	Glucose	−0.6 to 0.8	PhB/pH 7.0	0.0–8.0	10	[127]
Pt-Teflon/TTF-TCNQ—Si oil paste/AA	L-ascorbic acid	−0.1 to 3.3	PhB/pH 8.6	0.001–50	500	[108]
Pt/TTF-TCNQ/sulphite oxidase/Si oil paste	Sulphite oxidase (EC 1.8.3.1)	−0.1 to 0.3	PhB/pH 8.6	0.0056–0.15	1	[142]
Pt-Teflon/TTF-TCNQ/	Choline oxidase betaine\hypoxanthine xanthine	−1.5 to 3.5 (vs SCE)	PhB/pH 7.4	0.06–8.0 0.05–18 0.0–0.87 0.002–0.108	95∙10^3^ 275∙10^3^ 900∙10^3^ 8500	[109]
TCNQ/CP/NADH	Dihydronicotinamide adenine ainucleotide	−0.75 to 1.0	PhB/pH 7.0	0.0–70	10,000	[112]
Pt/NMP-TCNQ—PVC/GOx/PPD	Glucose	−0.2 to 0.4	PhB/pH 7.0	0.1–0.5	150	[107]
Pt/NMP-TCNQ/GOx/dialysemedbran	Glucose	−0.1 to 0.1 (vs. SCE)	PhB/pH 7.2	0–30	5000	[140]
TTF-TCNQ/CTC/SEC/GOx	Glucose	−0.2 to 0.35	PhB/pH 7.0	0.0–30	10,000	[26]
PPW/TTF-TCNQ/H_2_O_2_/GOx/heteropolypyrrole film	Glucose	−1.5 to 1.5 (vs SCE)	PhB/pH 7.0	1.6–10	1600–10,000	[27]
TTF-TCNQ/GOx-parafin oil-PIB	Glucose	−0.1 to 0.25	PhB/pH 7.0	0.0–100	0.2	[121]
Pt/TTF-TCNQ/SPE/FDH/TECOFLEX SG80	Formaldehyde	−0.2 to 0.3	PhB/pH 7.4	0.25–1.0	500	[143]
GA/CoPC/TCNQ/SPE/ AChE	Heavy metals (Cd^2+^)	0.01 to 0.15	AcB/ pH 5.1–5.5 PhB/ pH 6.3–7.0	2.5∙10^−3^–0.025	0.49	[136,137]
MWCNT-TCNQ/SPE/AChE/Nafion	AChE inhibitors	−0.4 to 0.8	PhB/pH 8.0	0.1–50	3∙10^−5^ 4∙10^−4^	[144]
Pt/TTF-TCNQ-IL/CP/AChE/	Carbamate drugs: Eserine Neostigmine	−0.4 to 0.8	PhB/pH 8.0	0.1–5.0	2.6∙10^−5^ 3∙10^−4^	[145]
Au/CNTs/TTF-TCNQ/A/Teflon Au/CNTs/TTF-TCNQ/DM/Teflon Au/CNTs/TTF-TCNQ/GDH/osmium redox polymer	Ascorbate Dopamine Glucose	0.37 to 0.7 0.0 to 0.6 (vs. NHE)	PhB/pH 7.0	0.1–5 0.5–1.5 0.1–5	1.029 1.48 100	[138]
Pt/TCNQ/SPE/AChE/polyaniline	Anticholinesterase pesticides	−0.05 to 1.7	PhB/pH 7.8	0.01–0.032	5	[146]
TCNQ/CP/HEC/AChE	Anticholinesterase pesticides	0.1	PhB/pH 8.0	2.5∙10^−6^–7.5∙10^−4^	0.2–1.5 ppb	[147]
Pt/GP/TTF-TCNQ/GOx	Glucose	−0.45 to 0.6	PhB/pH 7.5	1.8–3.6	100	[123]
Pt/TTF-TFCNQ/CP/O_2_/GSH	Glutathione in hemolysed erythrocyte	−0.015 to 0.25 (vs. SCE)	PhB/pH 7.8	0.005–0.340	0.3	[117]
Pt/CE/TTF-TCNQ/SPE/GLOx/Teflon	L-Glutamate	−0.1 to 0.5	ImB/pH 7.0	0.0–100	50	[155]
PVC/TTF–TCNQ/GOx/glutaraldehyde	Glucose	−0.15 to 0.4	PhB/pH 7.4	5–8	8.5–19	[156]
PVC/TTF-TCNQ-Au_coll_	Glucose	0.01 to 2.0	PhB/pH 7.4	0.1–2.0	6.2	[157]
TCNQ/EPG/AA	L-ascorbic acid	−0.325 to 0.025	PhB/pH 7.0	1–10	100	[109]
Pt/TCNQ/EPG/Nafion Pt/TCNQ-NPOE-PVC/EPG/THATPB/K ionophore II	Na+ K+	−0.079 to 0.237 −0.7 to 0.6	PhB/pH 3–10 PhB/pH 3–8	135–145 3.6–5.0	0.2 0.1	[148]
TCNQ/GO/GCE/GSH	Glutathione	−0.05 to 0.5	PhB/pH 7.2	2.5∙10^−4^–0.1243 and 0.1243–1.67	0.15	[50,158]
Pt/Co_3_O_4_/MWCNT-TCNQ-CHIT/GCE	Xanthine	−1.0 to 1.0	PhB/pH 7.0	1.9·10^−3^–0.12	0.36	[135]
CPE/TCNQ/AA CPE/TCNQ-PdNP-1/AA CPE/TCNQ-PdNP-2/AA	Ascorbic acid	0.1 to 0.3	PhB/pH 7.0	0.05–0.625	51.61 44.38 30.10	[149]
MWNT/[BMIM][PF_6_]-[TTF-TCNQ]/ [BMIM][PF_6_]/PPZ	Perphenazine	−0.4 to 0.6	PhB/pH 10.0	6·10^−5^–1.6·10^−2^	0.0205	[150]
TCNQ-Pd-Co@NC/CPE/NADH	NADH	−0.2 to 0.6	PhB/pH 7.0	0.01–0.25	5.17	[151]

Abbreviations: AA—ascorbic acid; AcB—acetate buffer; AChE—acetylcholinesterase; Au_coll_—colloidal gold; BMIM—1-Butyl-3-methylimidazole; CE—graphite electrode; CHIT—chitosan; CNTs—carbon nanotubes; CoPC—cobalt phthalocyanine; CPE—carbon paste electrode; DM—dialysis membrane; EPG—edge-plane pyrolytic graphite; FDH—formaldehyde dehydrogenase; GA—glutar-aldehyde; GCE—glassy carbon electrode; GDH—glucose dehydrogenase; GLOx—L-glutamate oxidase; GO—graphene oxide; GOx—glucose oxidaze; GSH—glutathione; HEC—hydroxyethyl-cellulose; IL—ionoc liquid; ImB—imidazole buffer; KB—carbon black electrode; K ionophore II—bis[(benzo-15-crown-4)-40-ylmethyl]-pimelate; LOD—limit of detection; LR—linearity range; MWCNT—multi-walled carbon nanotubes; NADH—nicotinamide adenine dinucleotide; Nafion—negatively charged perfluorosulfonated cation-exchange polimer; NC—nanocarbon; NHE—normal hydrogen electrode; NMP—phenazine methosulphate; NP—nanoparticle; NPOE—2-nitrophenyl octyl ether; PhB—phosphate buffer; PIB—poly iso-butylene; POx—peroxidase; PPD—poly-o-phenylenediamine; PPZ—perphenazine; PPW—platinized Pt wire; PVC—polyvinylchloride; SHE—standard hydrogen electrode; SPE—screen-printed electrode; THATPB—tetraheptylammonium tetraphenylborate.

**Table 2:** The major areas of applications of metal–TCNQ compounds.

M-TCNQ Compound	Applications	Refs.
Na/K/Rb/CsTCNQ	Electrode surfaces	[129]
KTCNQ	Organic electronics: optoelectronic switches and gas sensors	[186]
AgTCNQ	Organic electric bistable films for ultrafast electric memories; CT nanoelectrode; micro-/nanowires; current switch; surge voltage protectors and voltage sensors	[187,188,189,190]
CuTCNQ	Organic electric bistable films for ultrafast electric memories; micro-/nanowires; current switch; surge voltage protectors; voltage and pressure sensors; bistable material and prototypes of molecular electronics; nanorod and nanotube thermoelectric devices and new morphology of 1D nanocones; mediator of different analytes (glucose sensor); anode material for photoelectrochemical water oxidation	[25,188,189,192,193,194,195,196,197,198,205]
Co[TCNQ]_2_(H_2_O)_2_	Micro-/nanowires and crystalline thin films	[6,189]
Ca[TCNQ]_2_	1D/2D or 3D structured square/rectangular sheets	[202]
Pb(TCNQ)_2_	Effective surface area for catalytic reaction	[203]
Pb(TCNQF_4_)_2_	Good redox catalysts	[203]
Fe(TCNQ)_2_	3D electrodes for oxygen evolution reaction application	[204,205,206]
[M(N-N)_2_](TCNQ)_2_ (M = Mn, Fe, Co, Ni; N-N = phen, dips)	Ion-selective electrodes	[211]
Mn(TCNQ)_2_	3D catalyst electrodes for oxygen evolution reaction application	[212]
AlBe-TCNQ, BeSi-TCNQ; Al_2_TCNQ, B_2_TCNQ, Be_2_TCNQ	2D catalysts electrodes for electrocatalytic improving CO_2_ conversion	[216]
CoTCNQ	Electrodes for NO_2_ sensing	[217]

**Table 3:** TCNQ-based battery chemistries.

Battery Type	Features
Aqueous Li/Na/K/CuTCNQ	High safety nature and capacity, good charge/discharge performance, low cost, high cycle life and working voltage, low toxicity, super-high affinity to electrolyte
Li/F_4_TCNQ	High capacity and current density, high stability
Aqueous Zn/TCNQ	High safety and stability, good compatibility with water, rich abundance of zinc, environmental benignity of zinc
Zn/TTF-TCNQ	Initial high capacity, inherently high conductivity, superior high rate and cycling performance
Zn/TCNQ/FTCNQ/F_4_TCNQ	High safety and stability, good cycle life, high energy density
Aqueous Al/TCNQ@PPy	High cycling stability and rate performance, good reversibility and feasibility
NH_4_^+^/TCNQ-rGO	Large surface area, superior performance, high capacity, stable cycle life

## Data Availability

No new data were created or analyzed in this study.
